# Gypenoside L inhibits autophagic flux and induces cell death in human esophageal cancer cells through endoplasm reticulum stress-mediated Ca^2+^ release

**DOI:** 10.18632/oncotarget.10159

**Published:** 2016-06-18

**Authors:** Chenghui Liao, Kai Zheng, Yan Li, Hong Xu, Qiangrong Kang, Long Fan, Xiaopeng Hu, Zhe Jin, Yong Zeng, Xiaoli Kong, Jian Zhang, Xuli Wu, Haiqiang Wu, Lizhong Liu, Xiaohua Xiao, Yifei Wang, Zhendan He

**Affiliations:** ^1^ Department of Pharmacy, School of Medicine, Shenzhen Key Laboratory of Novel Natural Health Care Products, Innovation Platform for Natural Small Molecule Drugs, Engineering Laboratory of Shenzhen Natural Small Molecule Innovative Drugs, Shenzhen University, Shenzhen, China; ^2^ College of Life Science and Technology, Jinan University, Guangzhou, China; ^3^ The First Affiliated Hospital of Kunming Medical University, Kunming, China; ^4^ College of Life Sciences, Shenzhen University, Shenzhen, China; ^5^ The First Affiliated Hospital of School of Medicine, Shenzhen University, Shenzhen, China

**Keywords:** ROS, unfolded protein response, autophagic flux inhibition, vacuolation, Ca^2+^ release

## Abstract

Esophageal cancer is one of the leading cause of cancer mortality in the world. Due to the increased drug and radiation tolerance, it is urgent to develop novel anticancer agent that triggers nonapoptotic cell death to compensate for apoptosis resistance. In this study, we show that treatment with gypenoside L (Gyp-L), a saponin isolated from *Gynostemma pentaphyllum*, induced nonapoptotic, lysosome-associated cell death in human esophageal cancer cells. Gyp-L-induced cell death was associated with lysosomal swelling and autophagic flux inhibition. Mechanistic investigations revealed that through increasing the levels of intracellular reactive oxygen species (ROS), Gyp-L triggered protein ubiquitination and endoplasm reticulum (ER) stress response, leading to Ca^2+^ release from ER inositol trisphosphate receptor (IP_3_R)-operated stores and finally cell death. Interestingly, there existed a reciprocal positive-regulatory loop between Ca^2+^ release and ER stress in response to Gyp-L. In addition, protein synthesis was critical for Gyp-L-mediated ER stress and cell death. Taken together, this work suggested a novel therapeutic option by Gyp-L through the induction of an unconventional ROS-ER-Ca^2+^-mediated cell death in human esophageal cancer.

## INTRODUCTION

Esophageal cancer is the fifth leading cause of cancer death for male and eighth for female in developing countries [1]. The current therapeutic treatments are radiation therapy, chemotherapy, surgery and combinations of them [2, 3]. However, due to the poor diagnosis at an early phase, high recurrence of drug and radiation tolerance, developing novel and efficient chemotherapeutic agents for the prevention and treatment of esophageal cancer is emergently required.

Autophagy plays an important role in cancer development and therapy [4]. Tumor cells usurp autophagy to enhance their metastasis and survival in metabolic stress such as DNA damage and hypoxia [5–7]. Besides, autophagy is one of the mechanisms by which tumor cells acquire therapy resistance [8–10]. Indeed, several reports have demonstrated that inhibiting autophagic flux significantly induces cell death or enhances the sensitivity of tumor cells to chemotherapeutic agents [11–13]. Therefore, inhibition of autophagy pathway is an alternative strategy for cancer therapy [14].

Natural products are the important resources for drugs discovery of cancer therapy [15]. *Gynostemma pentaphyllum*, also known as “South ginseng” in china, has been extensively used in traditional human medicine or tea in Asia. Gypenosides (Gyp), saponins extracted from *G. pentaphyllum*, have shown a wide range of beneficial biological activities such as cholesterol-lowering, hypoglycemic, antioxidant and immune-potentiating effects [16–19]. Besides, fewer reports have described the anti-tumor activity of Gyp [20–22]. However, the specific functional component of Gyp and the detailed mechanism remain unclear.

**Figure 1:**
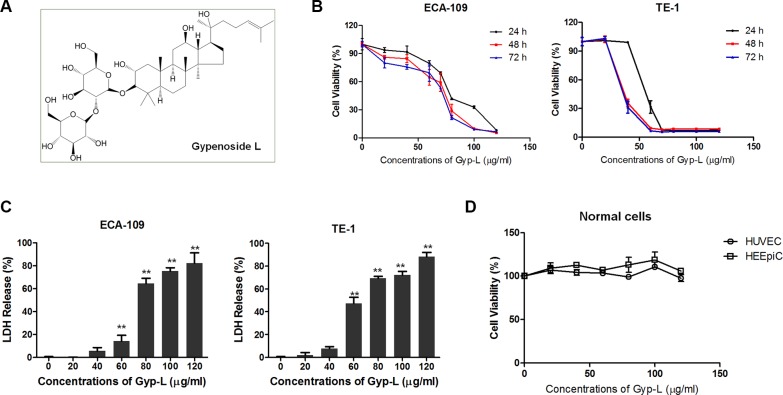
Gyp-L induces cell death (**A**) Chemical structure of Gyp-L. (**B**) Gyp-L inhibited the growth of esophageal cancer cells. ECA-109 and TE-1 cells were incubated with increasing doses of Gyp-L for 24 h, 48 h and 72 h, respectively. Cell viability was determined by MTT assay. Data are the mean ± SD of at least three independent experiments. (**C**) ECA-109 and TE-1 cells were treated with Gyp-L for 24 h, and LDH activity in the medium was measured as described in Materials and Methods. The necrosis was relatively compared with the results from total cell lysates as 100%. The result from 10% ethanol-treated cells for 24 h was used as necrosis positive control (P.C.). **P < 0.01 compared with the control group. (**D**) Normal cells HUVEC and HEEpiC cells were treated with Gyp-L for 24 h and cell viability was measured with the MTT assay.

In this study, we identified Gypenoside L (Gyp-L) (Figure 1A), a saponin isolated from Gyp (Supplementary Figure S1), possessing the anticancer activity in human esophageal cancer cells. Gyp-L was able to inhibit the growth of esophageal cancer cells, which was accompanied by lysosomal swelling and a halt in the execution of autophagy at the stage of autophagosome-lysosome fusion. Mechanically, Gyp-L-induced cell death required ROS-mediated unfolded protein response (UPR), and subsequently ER Ca^2+^ release. These results suggested Gyp-L as a novel therapeutic agent to induce an unconventional cell death in human esophageal cancer.

## RESULTS

### Gyp-L induces cell death in human esophageal cancer cells

To investigate the cytotoxicity of Gyp-L on human esophageal cancer cells, ECA-109 and TE-1 cells were treated with various concentrations of Gyp-L for 24 h, 48 h, 72 h and cell viability was determined by MTT assay (Figure 1B). Gyp-L exhibited potent growth inhibitory effect on both cancer cell lines in a dose-dependent manner. Besides, treatment with Gyp-L showed a significant increase in cell death, when it was measured by lactate dehydrogenase (LDH) release assay (Figure 1C). On the contrary, no cell death-inducing effect of Gyp-L on normal cells (HUVEC) or normal esophageal epithelial cells (HEEpiC) was observed (Figure 1D).

### Gyp-L induces cytoplasmic vacuolation and lysosomal swelling and fusion

**Figure 2:**
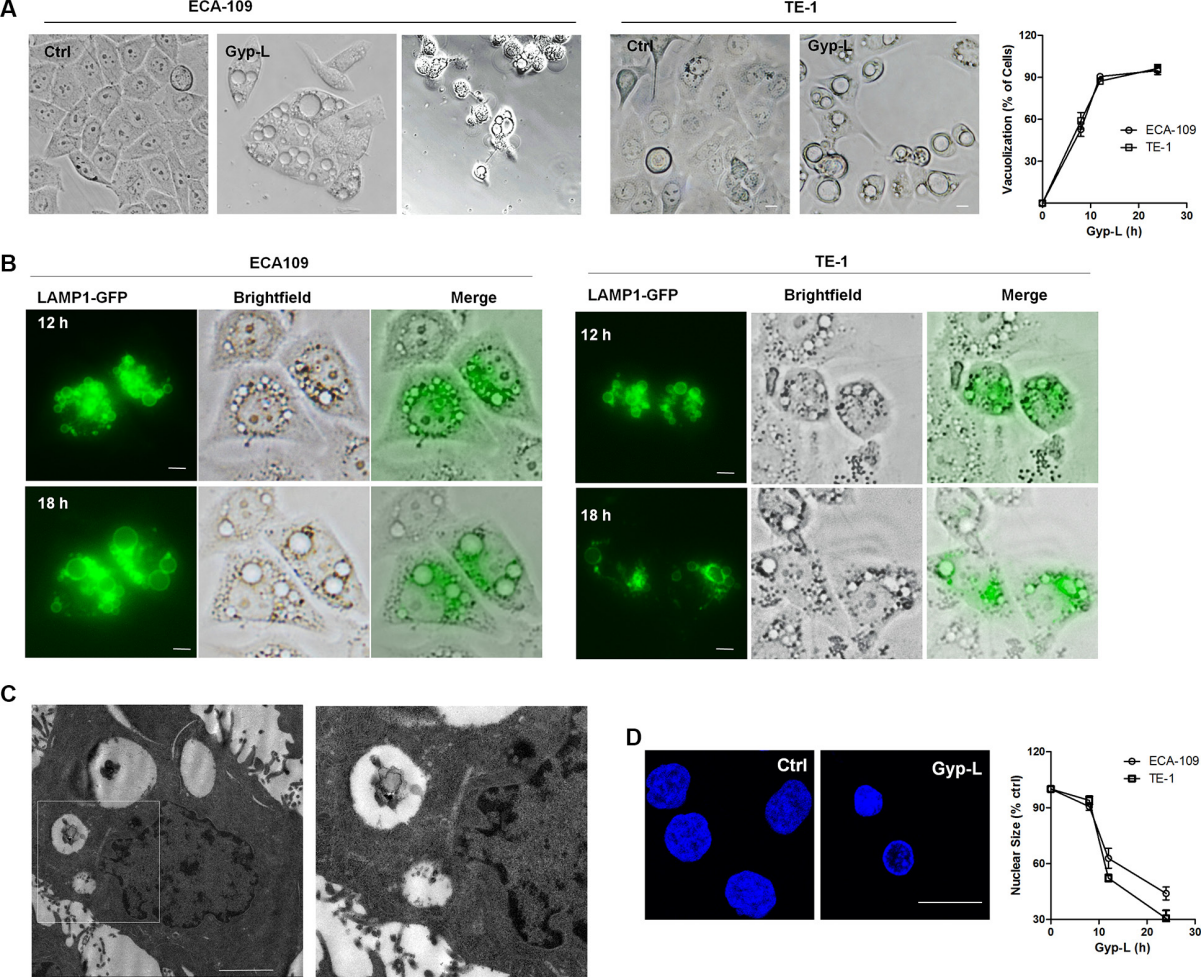
Gyp-L-induced cell death associates with lysosomal fusion and swelling (**A**) Gyp-L treatment induced cytoplasmic vacuolation. ECA-109 or TE-1 cells were treated with Gyp-L (60 μg/ml) for different times. Cell morphology was photographed under a microscopy (40 × magnification). Scale Bar: 20 μm. Right panel showed the quantification of the percentage of cells having visible vacuoles in ECA-109 and TE-1 cells upon Gyp-L (60 μg/ml) treatment for different times. (**B**) The cells were transfected with LAMP1-GFP for 24 h before treated with Gyp-L (60 μg/ml) for indicated times. (**C**) ECA-109 cells were treated with Gyp-L (60 μg/ml) for 24 h, fixed and examined using transmission electron microscopy. Higher power magnification of the image of Gyp-L-treated cells revealed lysosomes. Scale bar: 2 μm. (**D**) DAPI staining visualized the nucleus in medium- and Gyp-L-treated (12 h, 60 μg/ml) ECA-109 cells. Quantification of nuclear size of ECA-109 and TE-1 cells after 8- to 24-h treatment with Gyp-L (60 μg/ml).

The morphological changes were visualized and Gyp-L induced extensive cytoplasmic vacuolation, which affected ~95% of cells after 24 h (Figure 2A). Typically, the numbers of vacuoles reduced and the sizes increased at higher concentration. The vacuolated cells showed an intact nucleus, shrined at later time points and underwent cell death. Interestingly, lysosomal membrane marker LAMP1-GFP was found to localize at the edge of the cytoplasmic vacuoles (Supplementary Figure S2A and Figure 2B), indicating that these vacuoles were hypertrophic lysosomes. Through fluorescence microscopy assay using Lyso-Tracker Red, we also found that Gyp-L-induced vacuoles were colocalized with lysosomes (Supplementary Figure S2B). Following Gyp-L treatment, small lysosomes fused with each other and the sizes of the vacuoles increased largely (Figure 2B). Additionally, electron microscopic analysis showed that the ECA-109 cells treated with Gyp-L contained large vacuoles (Figure 2C), and higher magnification electron micrographs clearly showed the presence of partially degraded cytoplasmic materials in the vacuoles (Figure 2C, right panel). Due to the progressive vacuolar swelling upon treatment with Gyp-L, the nuclear size of ECA-109 or TE-1 cells was reduced by > 40% within 12 h (Figure 2D). In addition, although the LysoTracker signal as well as the level of red fluorescence of acridine orange (AO)-stained cells increased over the first 6 h of treatment with Gyp-L, the signal intensity of both dyes was decreased after 24 h of treatment. All the large vacuoles lost their red acridine orange signal (Supplementary Figure S2C), indicating that these dilated lysosomes lost functionality. Taken together, these results indicated that Gyp-L-induced swelling and dysfunction of lysosomes correlated with loss in cell viability

### Gyp-L-induced cell death is apoptosis-independent

**Figure 3:**
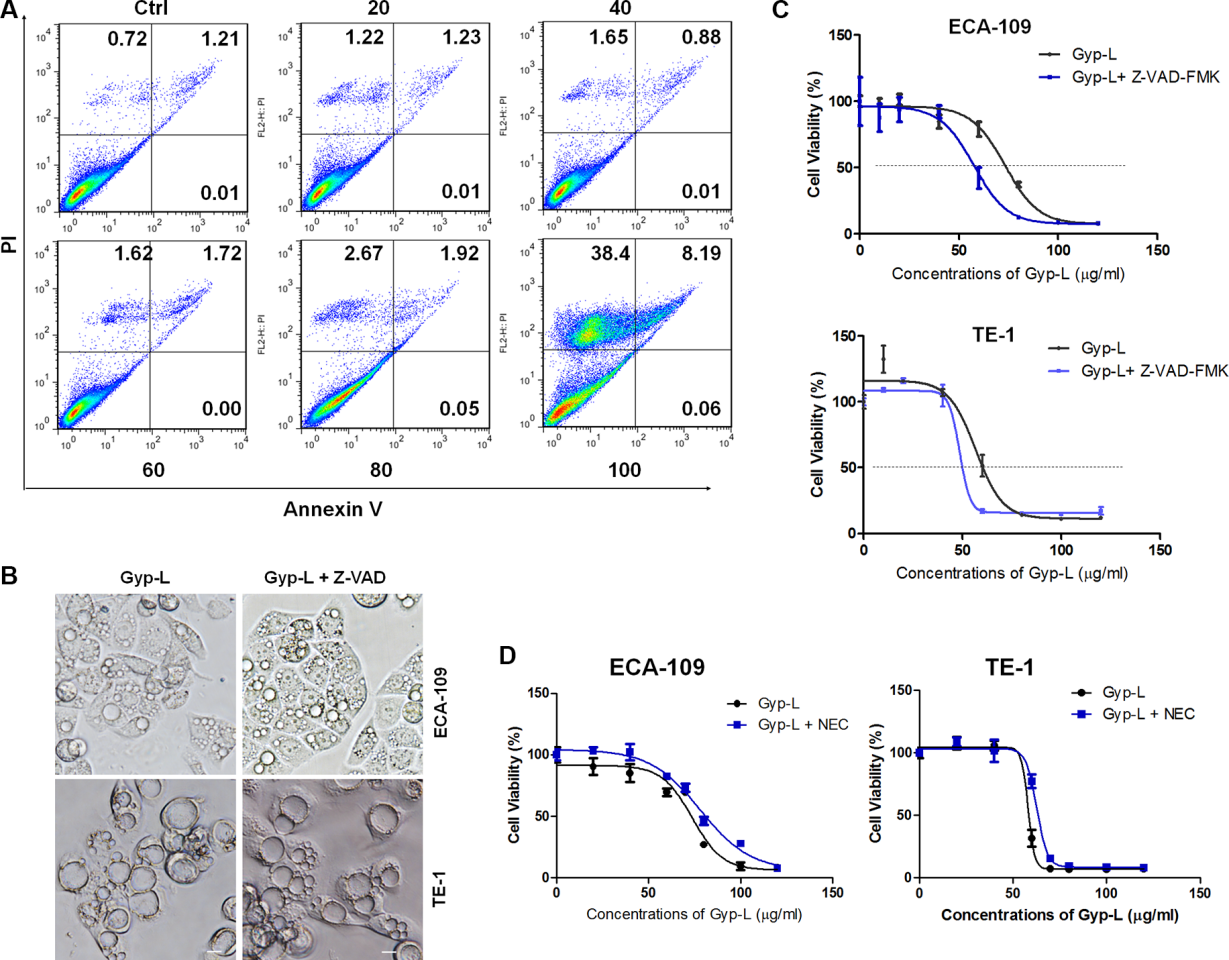
Gyp-L induces non-apoptotic cell death (**A**) Induction of apoptosis by Gyp-L in ECA-109 cells was determined by flow cytometry using Annexin-V-FITC/PI staining after treatment with Gyp-L for indicated concentrations for 24 h. Similar results were obtained in three independent experiments. (**B**–**C**) The effect of Z-VAD (50 μM) on Gyp-L-induced cell death and cytoplasmic vacuole formation. Scale bar: 20 μm. (**D**) ECA-109 or TE-1 cells were treated with Gyp-L in the presence or absence of NEC (20 μM) for 24 h. Cell viability was measured with the MTT assay.

To gain insight into the nature of Gyp-L-induced cell death, we examined the ratio of apoptosis using flow cytometry after Annexin V/PI double staining. As shown in Figure 3A, Gyp-L barely induced apoptosis, as most of the dead cells belonging to necrosis or other types of cell death. We then applied the pan-caspase inhibitor Z-VAD-FMK (Z-VAD) to Gyp-L treatments. Inclusion of Z-VAD-FMK in a non-cytotoxic concentration significantly inhibited caspase activity (Supplementary Figure S3A). However, Z-VAD-FMK prevented neither Gyp-L-medicated cell death (Figure 3B) nor cytoplasmic vacuolation (Figure 3C). In contrast, treatment with Z-VAD-FMK enhanced Gyp-L-induced cell death, suggesting that Z-VAD-FMK switches more apoptotic-liked cell death to Gyp-L-mediated cell death. Moreover, no cleaved-PARP, Caspase-3 or Caspase-9 were detected by western blot (data not shown). In addition, we measured mitochondrial membrane potential with JC-1 staining. We found that no changes occurred when ECA-109 cells were treated with Gyp-L for 24 h (Supplementary Figure S3B), further suggesting that Gyp-L induced cell death is apoptosis-independent. Furthermore, we assessed the effect of Necrostatin-1 (NEC), a necrosis inhibitor, on Gyp-L cytotoxicity. Unlike apoptosis, loss of necroptosis provided partial rescue of cell viability with Gyp-L treatment (Figure 3D). Moreover, cell cycle distribution was monitored using flow cytometry. Essentially, treatment with Gyp-L resulted in the accumulation of cells in the G2/M phase, with a concomitant reduction in the proportion of cells in the S phase (Supplementary Figure S3C). It is interesting to investigate the contribution of the hindered cell cycle progression to Gyp-L-induced cell death.

### Gyp-L regulates autophagy

**Figure 4:**
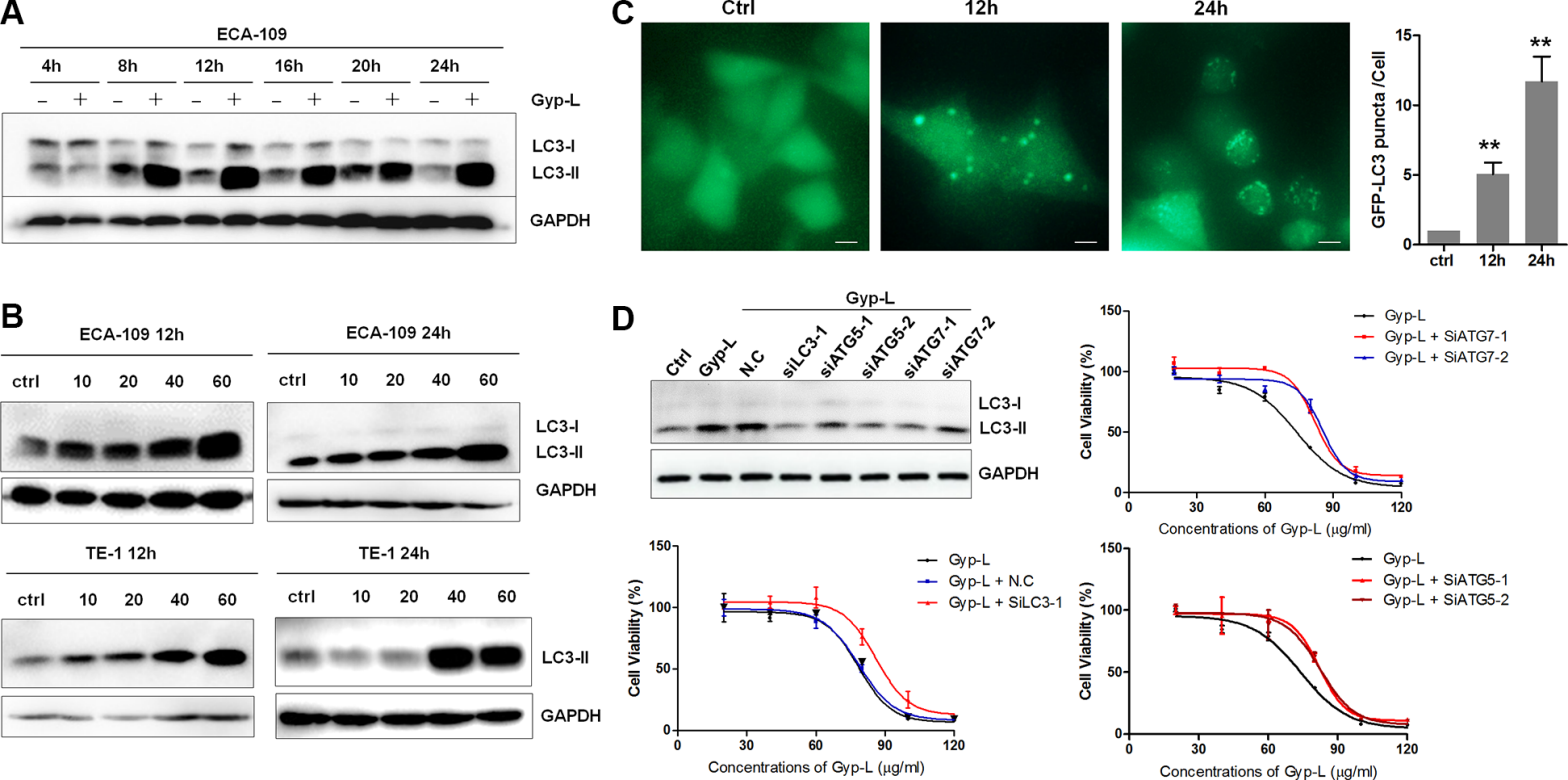
Autophagy is involved in Gyp-L-induced cell death (**A**) The time course of LC3-II protein expression induced by Gyp-L. ECA-109 cells were treated with 60 μg/ml Gyp-L for indicated times and the protein levels of LC3-II and GAPDH were analyzed by western blot assay. (**B**) ECA-109 and TE-1 cells were treated with different concentrations of Gyp-L for indicted times and cell lysates were subjected to western blot assay for p62 and GAPDH. (**C**) GFP-LC3 punctation assay. ECA-109 cells were transfected with plasmid GFP-LC3 (2 μg) for 24 h before adding Gyp-L for another 12 h or 24 h. Images were captured by fluorescence microscopy and the average number of GFP-LC3 puncta per cell were determined. At least 50 cells from 5 representative fields were counted in each independent experiment. Scale bar: 20 μm. (**D**) Knockdown of autophagy-related genes prevented Gyp-L-induced cell death. ECA-109 cells were transfected with siRNAs against *LC3*, *ATG5*, *ATG7* or control siRNA (N.C) for 24 h, and then treated with various concentrations of Gyp-L for another 24 h. Cell viability were determined by MTT assay and western blot result showed the knockdown efficacy. Data presented are representative of three independent experiments.

Based on the progressive vacuoles formation and dysfunction of lysosome, it is easy to envision that autophagy process is interrupted. We therefore examined the effect of Gyp-L on autophagy. Gyp-L augmented the level of endogenous LC3-II in a time-dependent manner in ECA-109 cells (Figure 4A). The accumulation of LC3-II induced by Gyp-L was also observed in a dose-dependent manner in both ECA-109 and TE-1 cells (Figure 4B). We further used ECA-109 cells transiently expressing GFP-tagged LC3 to confirm the autophagy modulatory effect of Gyp-L (Figure 4C). Apparently, Gyp-L induced a GFP-LC3 puncta increase and the number of puncta per cell was largely increased after a longer incubation with Gyp-L. Next siRNAs targeting autophagy-related genes were used to investigate the role of autophagy in Gyp-L-induced cell death (Supplementary Figure 4D). Suppression of autophagy by knockdown of *ATG5*, *ATG7* or *LC3* in ECA-109 cells significantly attenuated Gyp-L-induced cell death (Figure 4D and Supplementary Figure S4A). Finally, inhibition of autophagy by 3-MA (10 mM), a class III PtdIns3-kinase inhibitor that has been widely used as a pharmacological inhibitor in autophagy studies, unexpectedly did not affect Gyp-L-mediated LC3-II accumulation (Supplementary Figure S4B), cytoplasmic vacuolation (Supplementary Figure S4C), as well as cell death (Supplementary Figure S4D). Previous studies have demonstrated that 3-MA could inhibit both class III and class I PtdIns3-kinase and exhibited a dual role in modulation of autophagy (induction or inhibition) [23, 24]. Additionally, the synergism between 3-MA and chemotherapeutic drugs in esophageal cancer cells seems to be independent of autophagy [25]. Therefore caution should be exercised in the application of 3-MA in cancer combinatory study. Together, these above findings suggested that Gyp-L-induced cytotoxicity depends on the autophagic pathway.

### Gyp-L inhibits autophagic flux

**Figure 5:**
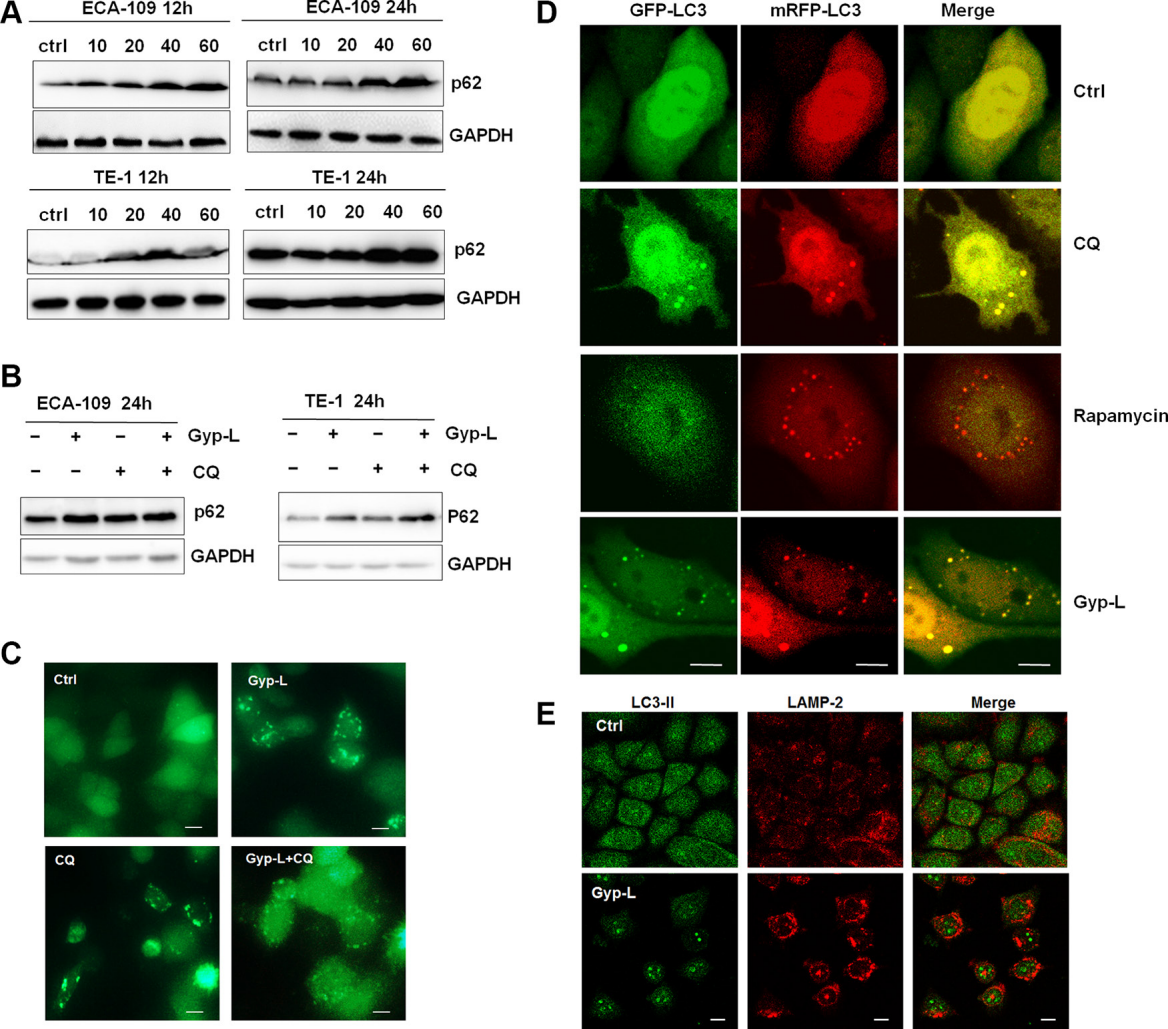
Gyp-L inhibits autophagic flux (**A**) treatment with Gyp-L led to the accumulation of autophagic substrate p62. ECA-109 and TE-1 cells were treated with different concentrations of Gyp-L for indicted times and cell lysates were subjected to western blot assay. (**B**–**C**) Inclusion of CQ (20 μM) enhanced the protein level of Gyp-L-induced LC3-II and GFP-LC3 punctation. (**D**) ECA-109 cells were transfected with mRFP-GFP-LC3 plasmid (2 μg). Twenty-four hours after the transfection, the cells were treated with 60 μg/ml Ro or 20 μM CQ or 10 μM Rapamycin for 12 h. Cells were then fixed and subjected to confocal microscopy. Scale bar: 10 μm. (**E**) Confocal microscopy showing the colocalization between LAMP2 and LC3. The cells were treated with Gyp-L for 24 h, fixed, and then stained with anti-LAMP2 (Red) and anti-LC3B (Green).

The increment of GFP-LC3 puncta or LC3-II level may results from the increased autophagosomes generation or the blockage of autophagosome-lysosome fusion process. However, the progressive vacuolation and lysosomal swelling upon Gyp-L treatment suggested that autophagy may be halted at the stage of autophagosomal-lysosomal fusion. To explore this possibility further, we performed an autophagic flux assay by measuring the total cellular amount of p62 to distinguish Gyp-L-mediated LC3 increment. p62 is degraded by lysosomal enzymes after autophagosome fuses with lysosome. As p62 is an autophagy substrate, increased autophagy levels are associated with p62 clearance. Consistently, immunoblot analysis showed that a remarkable increase of p62 was induced by Gyp-L in a time- and dose-dependent manner (Figure 5A). Adding of chloroquine (CQ), an autophagic inhibitor that can significantly increase the amount of LC3-II and p62 [26], not significantly enhanced the accumulation of p62 (Figure 5B), as well as GFP-LC3 puncta induced by Gyp-L (Figure 5C). Except for p62-mediated protein degradation in autophagy, malfunctioning mitochondria is also selectively targeted for autophagic degradation. According to this, we suspected whether impaired autophagic flux by Gyp-L may interrupt mitochondrial homeostasis. Actually, we used Mito-Tracker Green to measure mitochondrial mass and found a significant increase in Mito-Tracker Green fluorescence in both cells after 12 h exposure of Gyp-L (Supplementary Figure S5A). Together, these findings suggest that autophagy flux is inhibited by Gyp-L. Following this, we explored if autophagosome-lysosome fusion was impaired. To do this, tfLC3, a novel reporter protein (a chimeric LC3 protein fused tandemly with mRFP and GFP, mRFP-GFP-LC3), was used to monitor the autophagy process during Gyp-L treatment. GFP and RFP exhibit differing pH sensitivity, wherein GFP is quantitatively quenched in acidic compartments while RFP is stable. Therefore tfLC3 develops yellow dots (GFP-positive/RFP-positive) when localized to autophagosomes, and develops red dots (GFP-negative/RFP-positive) when localized to autolysosomes. As shown in Figure 5D, the number of yellow and red puncta increased in Rapamycin-treated cells, indicating that autophagic flux had increased. In the presence of Gyp-L, we observed an enhanced formation of yellow puncta without any significant increase in the number of red puncta, as well as CQ-treated cells. Additionally, the proteins LAMP2 and LC3B also did not colocalize in Gyp-L-treated cells, indicative of an arrest in the autophagic process at the stage of autophagosome-lysosome fusion (Figure 5E). Therefore, these above results clearly suggested that Gyp-L blocks the fusion between autophagosomes and lysosomes.

More importantly, CQ enhanced the cytotoxicity of Gyp-L on all esophageal cancer cells (Supplementary Figure S5B), suggesting that CQ and Gyp-L might have different mechanisms to impair the autophagosome-lysosome fusion process. Furthermore, we analyzed the mRNA expression of several lysosomal positioning proteins that have been demonstrated to regulate autophagysosome-lysosome fusion, including *RAB5*, *RAB7*, *RAB11* and *HDAC6* [27–29]. Treatment with Gyp-L significantly up-regulated the expression of *RAB5*, whereas no change was occurred with the expression of other genes (Supplementary Figure S5C). However, western blot assay showed that the protein level of RAB5 did not affected by Gyp-L (Supplementary Figure S5D). Besides, knockdown of RAB5 using siRNA has no effect on Gyp-L-induced cell death (Supplementary Figure S5E).

### Upregulation of protein ubiquitination and ER stress by Gyp-L

**Figure 6:**
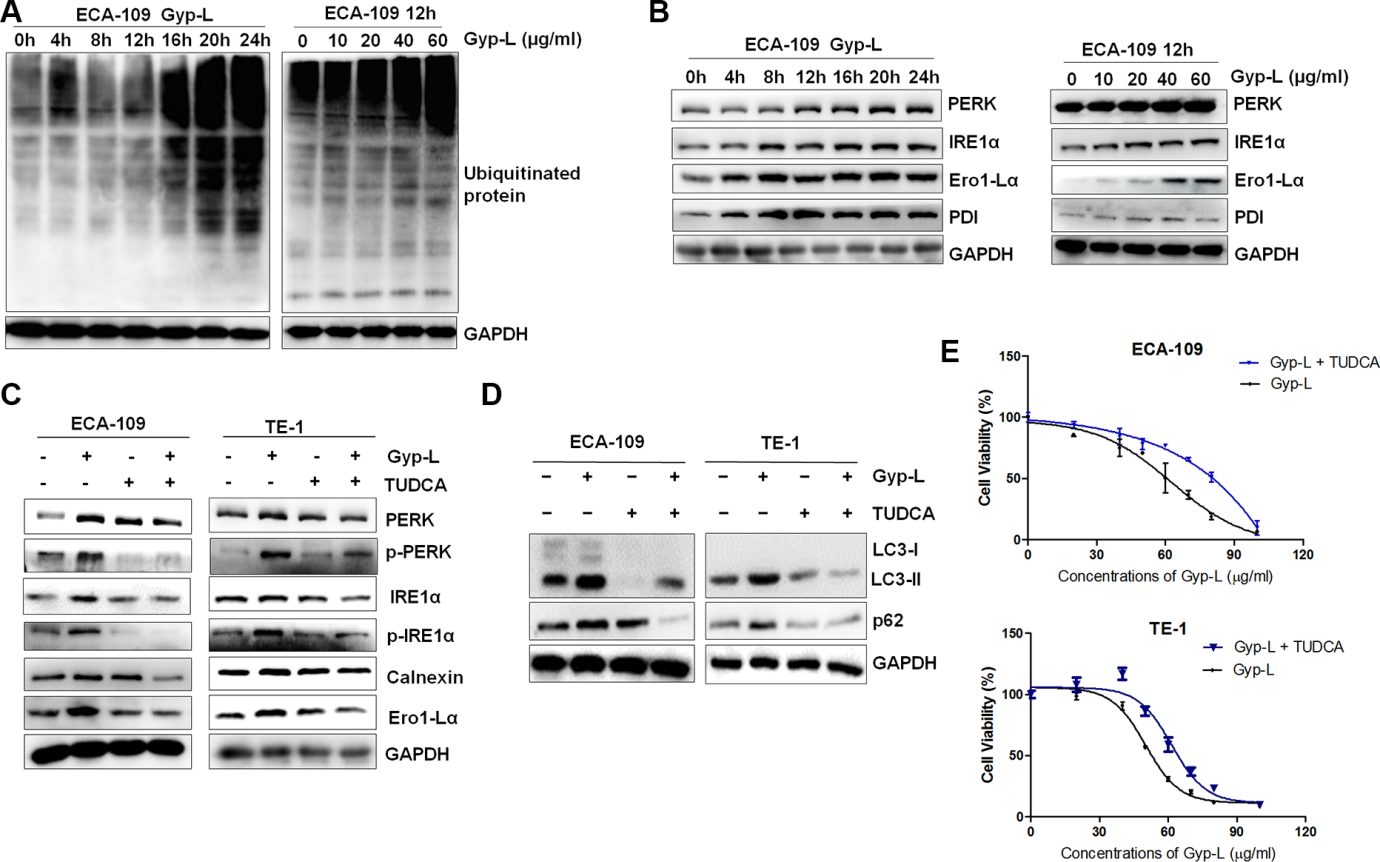
Protein ubiquitination and UPR activation by Gyp-L (**A**) Gyp-L time- and dose-dependently increased the levels of ubiquitinated proteins. ECA-109 cells were incubated with 60 μg/ml Gyp-L for indicated times, or treated with 0, 20, 40, 60 and 80 μg/ml Gyp-L for 12 h, and cell lysates were subjected to western blot. (**B**) Gyp-L activated the UPR pathway in a dose- and time-dependent manner. (**C**–**D**) TUDCA attenuated Gyp-L-mediated UPR pathway activation and autophagic flux inhibition. ECA-109 and TE-1 cells were treated with Gyp-L (60 μg/ml) in the presence or absence of TUDCA (40 μM) for 12 h and cell lysates were analyzed by western blot. (**E**) TUDCA protected cancer cells from Gyp-L-induced cytotoxicity. After 24 h treatment with Gyp-L in the presence or absence of TUDCA, cell viability was measured with the MTT assay. Data presented are representative of three independent experiments. Data are presented as the means ± SD or standard error of three independent experiments.

To understand the central mechanism through which Gyp-L inhibits autophagic flux and induces lysosome-associated cell death, we analyzed the possible role for ER stress in Gyp-L-promoted cell death. Since the accumulation of polyubiquitinated proteins is a characteristic of ER stress, we first assessed the level of total polyubiquitinated proteins. Western blot analysis showed that Gyp-L increased polyubiquitinated protein levels in a time- and dose-dependent manner (Figure 6A). To mitigate the accumulation of unfolded or misfolded proteins, stressed cells may activate a homeostatic intracellular signaling network cumulatively, the UPR pathway, to orchestrate the recuperation of ER function. As expected, the protein levels of several UPR associated proteins were largely increased in a time and dose-dependent way after Gyp-L treatment (Figure 6B). In addition, TUDCA, an specific ER stress inhibitor, significantly reduced Gyp-L-mediated activation of UPR (Figure 6C) and LC3-II accumulation (Figure 6D). Consistently, TUDCA apparently attenuated Gyp-L-mediated cell death in both ECA-109 cells and TE-1 cells (Figure 6E). These results indicated that ER stress plays a crucial role in Gyp-L-induced autophagic flux inhibition and cell death of esophageal cancer cells.

Furthermore, we examined the selectivity of Gyp-L and tested whether Gyp-L induced ER stress in normal esophageal epithelial cells. To our surprise, although Gyp-L induced cytoplasmic vacuolation in higher concentration in HEEpiC cells, only limited cells had vacuole formation and the percentage of cell with vacuoles was largely reduced (Supplementary Figure S6A). Besides, Gyp-L failed to significant induce the accumulation of LC3-II and p62 (Supplementary Figure S6B). Gyp-L also failed to activate ER stress in HEEpiC cells (Supplementary Figure S6C). Together, these results suggested that Gyp-L exhibits selectivity toward cancer cells to stimulate non-apoptotic cell death.

### ER calcium release potentiates the ER stress and cell death caused by Gyp-L

**Figure 7:**
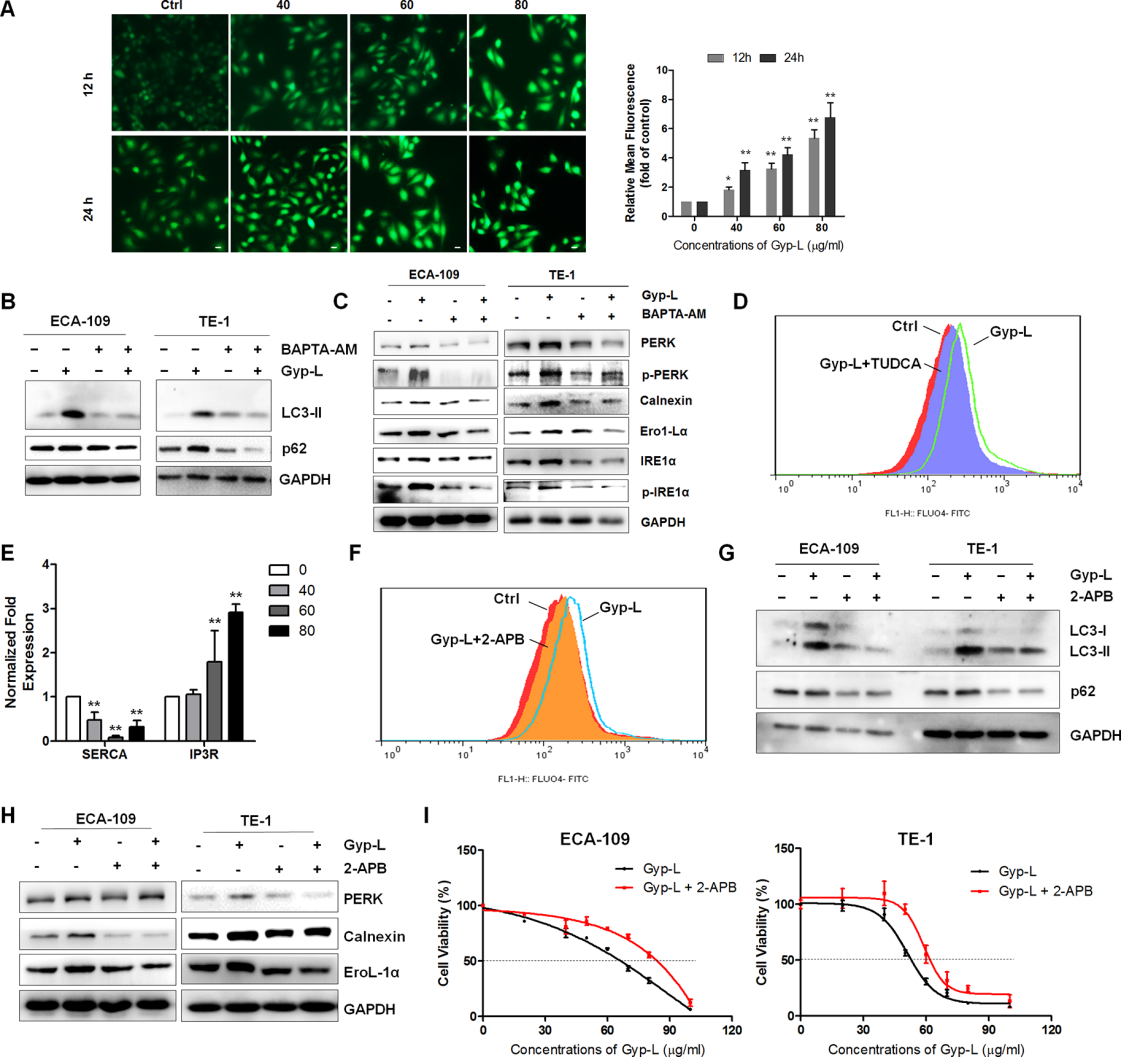
Ca^2+^ release from ER accelerates Gyp-L-induced ER stress and cell death (**A**) Gyp-L increased intracellular Ca^2+^ concentration. ECA-109 cells were treated with 40, 60 or 80 μg/ml Gyp-L for 12 h or 24 h, and cells were then incubated with 5 μM Fluo-4/AM for 60 min and detected by fluorescence microscopy. Left panel: Representative photomicrographs. Right panel: Quantification of Ca^2+^ production according to Fluo-4/AM fluorescence. Data are presented as the means ± SD. **P < 0.01 compared with the control group. (**B**–**C**) BAPTA-AM reduced LC3-II and p62 levels and the UPR pathway induced by Gyp-L. ECA-109 and TE-1 cells were treated with Gyp-L (60 μg/ml) in the presence or absence of BAPTA-AM (10 μM) for 12 h and cell lysates were analyzed by western blot. (**D**) TUDCA reduced Ca^2+^ levels. After treatment with Gyp-L and TUDCA for 12 h, ECA-109 cells were stained with Fluo-4/AM and the intracellular Ca^2+^ was measured by flow cytometry. (**E**) Expression of ER Ca^2+^ channels. (**F**–**I**) 2-APB reduced Ca^2+^ release from ER, the UPR pathway activation and LC3-II and p62 accumulation, as well as Gyp-L-induced cell death. Cells were treated with or without 2-APB (20 μM). Cell viability after 24 h treatment was determined by MTT assay.

ER stress is accompanied by alteration in Ca^2+^ homeostasis, and the ER Ca^2+^ store depletion by itself can induce ER stress and cell death [30]. We next sought to investigate whether Gyp-L perturbed intracellular Ca^2+^ homeostasis. ECA-109 cells were challenged with different concentrations of Gyp-L for 12 h or 24 h, and stained with Ca^2+^ specific indicator Furo-4/AM. Indeed, incubation with Gyp-L dramatically increased the intracellular Ca^2+^ levels (Figure 7A). An examination of the functional significance of this increase in Ca^2+^ levels showed that BAPTA-AM, a cell-permeable acetoxymethyl ester of the Ca^2+^ scavenger, inhibited Gyp-L-induced LC3-II accumulation (Figure 7B) and UPR activation (Figure 7C), suggesting that the increase in intracellular Ca^2+^ levels is critically associated with ER stress and cell death. Surprisingly, inhibiting ER stress by TUDCA apparently reduced intracellular Ca^2+^ concentration (Figure 7D), giving a hint that ER stress triggers the Ca^2+^ increment. Therefore, there existed a positive interplay between Ca^2+^ and ER stress.

Next, we explored the sources of the increased intracellular Ca^2+^ following Gyp-L treatment. The ER is a major reservoir of intracellular Ca^2+^ and several Ca^2+^ channels, including inositol trisphosphate (IP_3_) receptor (IP_3_R) and Sarco/endoplasmic reticulum Ca^2+^-ATPase (SERCA), control the efflux or influx of Ca^2+^ [30, 31]. Release of Ca^2+^ from the ER is mainly via IP_3_R while SERCA transfers Ca^2+^ from the cytosol to the lumen of the ER at the expense of ATP hydrolysis. Firstly, we found that treatment with Gyp-L significantly reduced the mRNA expression of *SERCA*, whereas *IP*_3_ *R* expression was largely increased (Figure 7E), indicating that IP_3_R may be responsible for Gyp-L-mediated intracellular Ca^2+^ increment. Then we used 2-APB, a selective inhibitor of IP_3_Rs, to investigate the role of IP_3_R in Ca^2+^ signaling. We found that pretreatment with 2-APB significantly decreased intracellular Ca^2+^ concentration (Figure 7F), and inhibited Gyp-L-induced LC3-II accumulation (Figure 7G), UPR activation (Figure 7H) and cell death (Figure 7I). Collectively, these results suggested that Ca^2+^ release from IP_3_R-operated stores potentiates the ER stress and contributes to Gyp-L-induced cell death in esophageal cancer cells.

### Gyp-L-induced ER stress and cell death requires new protein synthesis

**Figure 8:**
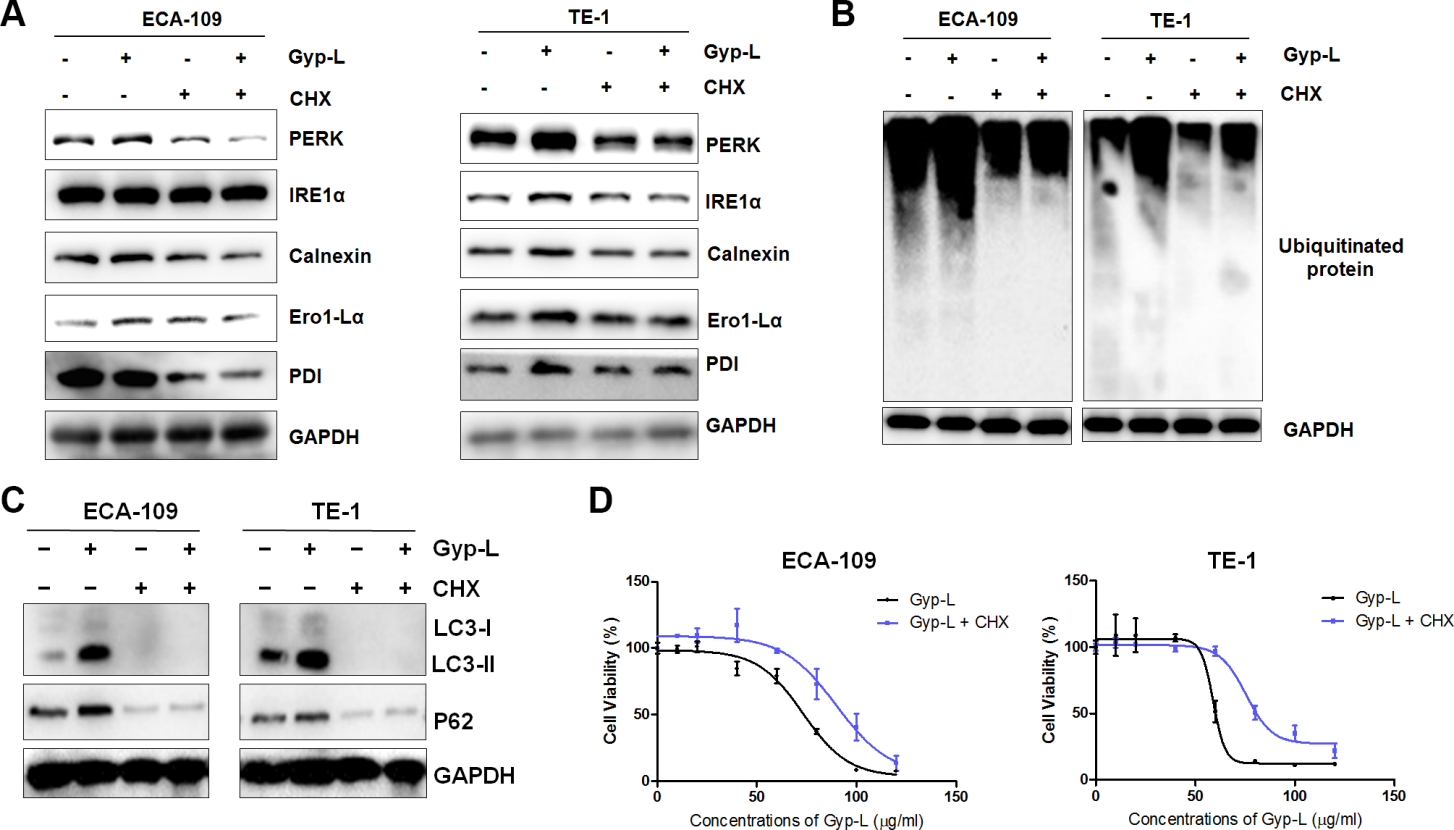
Protein synthesis contributes to Gyp-L-induced ER stress and cell death (**A**–**B**) Western blots showing the effect of CHX on the Gyp-L-induced UPR pathway activation and ubiquitinated proteins. ECA-109 and TE-1 cells were treated with Gyp-L (60 μg/ml) in the presence or absence of CHX (5 μg/ml) for 12 h and cell lysates were analyzed by western blot. (**C**–**D**) CHX reversed the inhibitory effect of Gyp-L on autophagic flux inhibition and reduced the cytotoxicity of Gyp-L on esophageal cancer cells.

Overload of protein in the ER-lumen could lead to the ER stress and subsequent cell death, thus we argued that abrogating synthesis of protein could exert a contrary role. Indeed, preventing protein synthesis by addition of cycloheximide (CHX) inhibited Gyp-L-induced ER stress in ECA-109 cells and TE-1 cells (Figure 8A). Treatment with CHX also significantly attenuated Gyp-L-induced protein ubiquitination (Figure 8B). In addition, CHX prevented LC3-II and p62 accumulation (Figure 8C), as well as Gyp-L-induced cell death (Figure 8D). These results revealed that Gyp-L-induced extensive misfolding of new synthesized proteins lead to ER stress and cell death.

### ROS generation critically contributes to the ER stress and cell death

**Figure 9:**
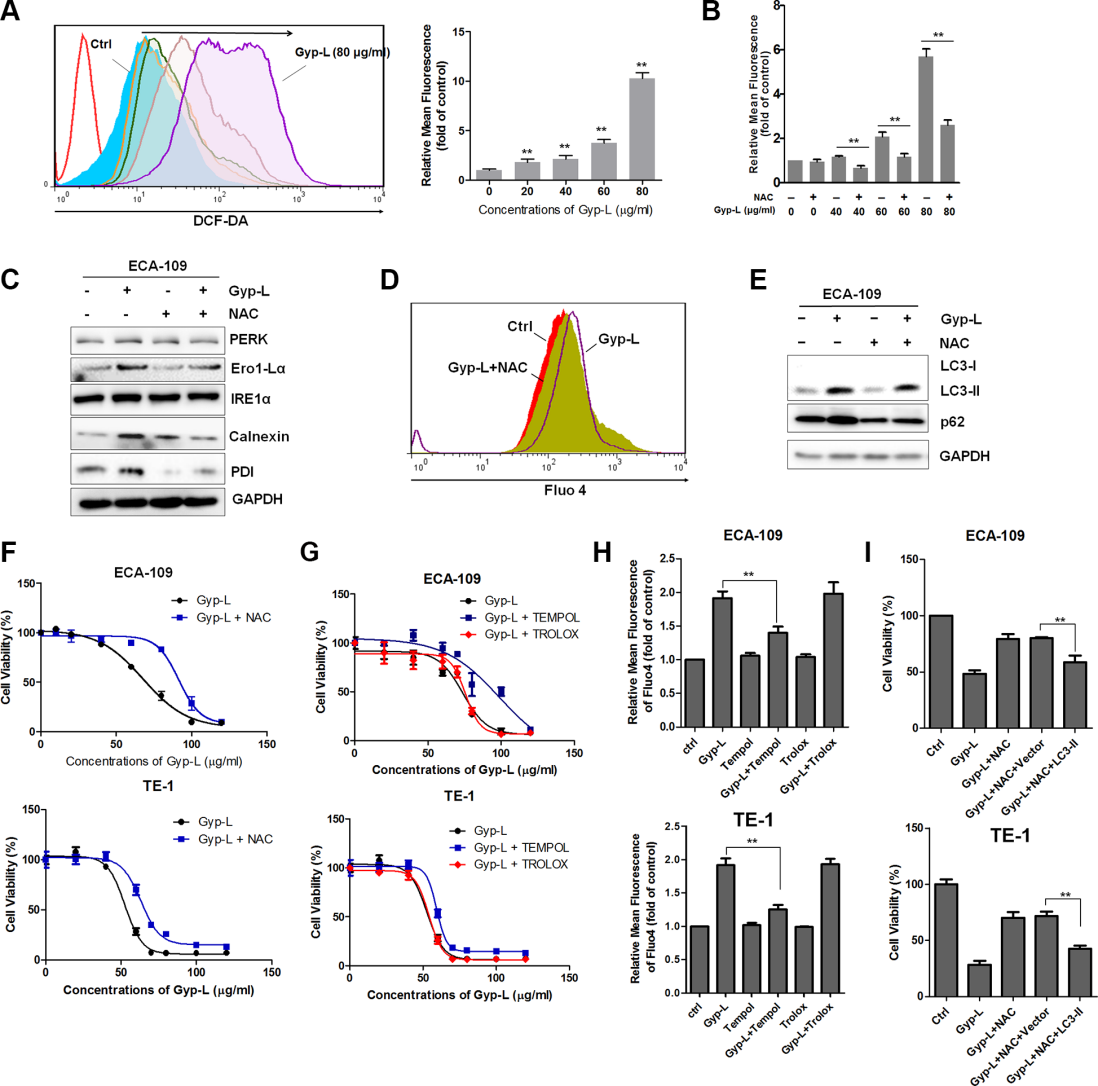
Gyp-L-induced cytotoxicity is dependent on intracellular ROS generation (**A**–**B**) Intracellular ROS generation induced by Gyp-L was measured in the absence or presence of NAC. ECA-109 cells were treated with or without NAC (5 mM) for 12 h before staining with DCFH-DA (10 μM) for 0.5 h. Intracellular ROS was measured by flow cytometry. Data presented are representative of three independent experiments. (**C**–**E**) NAC significantly reversed the activation of ER stress, Ca^2+^ release and autophagic flux inhibition induced by Gyp-L. (**F**) Blocking of ROS generation abolished the cytotoxicity of Gyp-L. (**G**–**H**) Effect of TEMPOL (2 mM) or Trolox (1 mM) on Gyp-L cytotoxicity and Gyp-L-induced Ca^2+^ release. (**I**) Overexpression of LC3-II ameliorated NAC-mediated inhibition. Cells were transfected with GFP-LC3 for 24 h and then treated with Gyp-L with or without NAC for 24 h.

It has previously been shown that ROS is involved in ER stress and cell death [32–34]. In order to further test this, we next examined whether Gyp-L also generates ROS in esophageal cancer cells and, if so, whether this contributes to the ER stress and subsequent cell death. Flow cytometry using a ROS fluorescent probe 2′,7′-dichlorofluorescein diacetate (DCF-DA) demonstrated that production of ROS was markedly increased after treatment with Gyp-L for 4 h (Supplementary Figure 9A). Consistently, pretreating the cells with NAC, a ROS scavenger, significantly decreased the production of ROS (Figure 9B). In addition, NAC pretreatment reversed Gyp-L-induced increment of several ER stress proteins (Figure 9C) and the intracellular Ca^2+^ concentration (Figure 9D). Vacuole formation and accumulation of LC3-II and p62 were also clearly reduced. (Supplementary Figure S7 and Figure 9E). Consequently, NAC pretreatment significantly ameliorated Gyp-L-mediated reduction in cell viability (Figure 9F). Furthermore, we tested the effects of other ROS scavengers, including TEMPOL, a superoxide dismutase mimetic, and Trolox, a vitamin E analog, on Gyp-L-induced cytoplasmic vacuolation and cell death. Surprisingly, only TEMPOL was capable to block Gyp-L-induced cell death (Figure 9G), Ca^2+^ release (Figure 9G) and cytoplasmic vacuolation (Supplementary Figure S7). Further works are required to investigate the different effects of TEMPOL and Trolox. Finally, overexpression of LC3 attenuated NAC-mediated cell death inhibition (Figure 9I). Taken together, these findings all clearly indicated that ROS generation acts as a upstream to trigger Gyp-L-induced ER stress and subsequent Ca^2+^ homeostasis and cell death.

## DISCUSSION

In the present work, we show that Gyp-L inhibits autophagic flux and induces nonapoptotic, lysosome-associated cell death in human esophageal cancer cells (Supplementary Figure S8). Exposure to Gyp-L was characterized by lysosomal swelling, autophagosome formation and halted autophagosome-lysosome fusion, ultimately leading to cell death. More detailed studies found that Gyp-L initially stimulated the generation of ROS, which subsequently triggered ER stress and Ca^2+^ release from IP_3_R-operated stores. Our data provide a preliminary insight into the possible use of Gyp-L as an anticancer drug for esophageal cancer therapy.

Compounds from natural plants are important sources for cancer therapeutic drug discovery. Gyp-L has been demonstrated to act as a potential activators of AMP-activated protein kinase [35, 36]. Previous study by Piao *et al.* has simply explored the anticancer activity of Gyp-L in human lung cancer cells [37]. However, the precise mechanism of Gyp-L-induced cell death have not been clearly determined. Herein we provided evidence that Gyp-L-induced cell death correlated with an increase in levels of autophagosomes in human esophageal cancer cells. In line with this, silencing of autophagy-related genes (*ATG5*, *ATG7* and *LC3*) protected against Gyp-L cytotoxicity (Figure 4D). However, treatment of Gyp-L resulted in a halt of the execution of autophagy at the stage of autophagosome-lysosome fusion, leading to hallmark accumulation of LC3B-II and p62 (Figure 5). Currently, disruption of the late stages of autophagy might lead to excessive accumulation of autophagic vacuoles containing deleterious undegraded material, which have the potential to turn autophagy into a destructive process [38, 39]. Such inhibition of autophagosome-lysosome fusion has previously been reported for the lysosomal inhibitor CQ, leading to vacuolization and lysosomal swelling as identified here for Gyp-L [40]. Therefore, Gyp-L seems to act as a lysosomal inhibitor to exert its anticancer activity by inhibiting the autophagic degradation of cytotoxic and deleterious materials.

Besides, Gyp-L-induced cytoplasmic vacuolation associated with increased LC3-II accumulation is similar to previous descriptions in colon, breast, glioblastoma cancer cell lines and mantle cell lymphoma [41–43]. However, in those studies large vacuoles derived from ER and mitochondrial whereas Gyp-L-induced vacuoles belonged to lysosomes. Besides, different signaling pathways were involved in cytoplasmic vacuolation and types of cell death were described inconsistently. For example, insulin-like growth factor-1 receptor-induced vacuolation showed to be mediated by the MAPK cascade and sensitive to the MEK inhibitor [44, 45]. 15d-PGJ2-induced vacuoles formation was mediated by both the MEK-1 and -2 cascades [41]. We showed here that Gyp-L treatment triggered the generation of ROS and UPR response. This process has also been observed to be associated with cytoplasmic vacuole formation and with enhanced antitumor activity [46]. Compared to normal cells, cancer cells appears to be a defect in their ability to eliminate Gyp-L-induced misfolded proteins as evident from the absence of activated ER stress in normal epithelial cells (Supplementary Figure S6C). These defects ultimately led to the accumulation of misfolded protein aggregates and novel non-apoptotic death. Moreover, treatment with Gyp-L did not negatively affect primary culture of normal epithelial cells. This differential sensitivity of normal versus malignant epithelial cells to Gyp-L provides a promising therapeutic window.

In addition, we showed that Gyp-L-induced ER stress triggered the release of Ca^2+^ from the ER, which was consistent with previous reports [47]. Indeed, intracellular Ca^2+^ chelating agent BAPTA-AM attenuated Gyp-L-induced UPR activation (Figure 7C), as well as Gyp-L-induced autophagic flux inhibition (Figure 7B). This result was further supported by the observation that following ER stress, BAPTA-AM could block autophagy flux induced by proteasome inhibitors [48]. One possible explanation is that depletion of cytosolic Ca^2+^ might interrupt the lysosomal function essential for complete autophagy flux [48]. Interestingly, the ER-Ca^2+^-mediated autophagic flux inhibition was similar with the effect of thapsigargin, a SERCA agonist that depletes ER Ca^2+^ reservoir [49–51]. Treatment with thapsigargin increased intracellular Ca^2+^, which in turn, blocked autophagy flux at the end stage by inhibiting the fusion of matured autophagosomes with lysosomes [50], or by preventing the closure of elongating phagophores [51]. It is therefore interesting to test the effect of Gyp-L on lysosome maturation and elongation. Together, these findings strongly suggest that autophagic flux inhibition is predominantly a consequence of ER-Ca^2+^ perturbation.

In conclusion, our results demonstrated that Gyp-L induced a nonapoptotic, lysosome-associated cell death in human esophageal cancer cells through ROS-ER-Ca^2+^ signaling. Our data suggest that small chemicals modulating autophagy and lysosomal function can serve as an alternative therapeutic option in esophageal cancer therapy, especially in apoptosis-resistant cancer treatment.

## MATERIALS AND METHODS

### Cell lines and cell culture

ECA-109 cells (TCHu 69) and TE-1 cells (TCHu 89), purchased from the Cell Bank of China Science Academy (Shanghai, China), and normal esophageal epithelia cells HEEpiC (purchased from Sciencell, 2720) were cultured in RPMI 1640 (Gibco) supplemented with 10% fetal bovine serum (FBS) (Gibco) and 1% penicillin/streptomycin (Invitrogen) at 37°C in a humid atmosphere with 5% CO_2_. HUVEC cells (CRL-1730, ATCC) were cultured in Dulbecco's Modified Eagle's Medium (DMEM) (Gibco).

### Chemicals and reagents

BAPTA-AM (B018) was purchased from Dojindo. CQ (C6628), TUDCA (T0557), 2-APB (D9754), CHX (C7698) and NAC (A9165) were purchased from Sigma-Aldrich. Z-VAD-FMK (S7023), Necrostatin-1 (S8037), Rapamycin (S1039) and TEMPOL (S2910) and 3-MA (S2767) were purchased from Selleck. Trolox (H828379) was purchased from Macklin.

Anti-LC3B rabbit polyclonal (L7543) and anti-p62/SQSTM1 antibody (P0067) were supplied by Sigma-Aldrich. Anti-LAMP2 (ab25631) and anti-p-IRE1α (ab48187) were purchased from Abcam. Anti-calnexin (2679), anti-p-PERK (3179),, anti-Ero1-Lα (3264), anti-IRE1α (3294), anti-RAB5 (3547), anti-PDI (3501), anti-PERK (5683), anti-Ubiquitin (3936), anti-GAPDH (5174), anti-caspase 3 (9662), anti-cleaved caspase 3 (9664), anti-caspase 9 (9504), anti-PARP (9542), anti-mouse IgG (7076) and anti-rabbit IgG (7074), HRP-linked antibodies were purchased from Cell signaling Technology. All inhibitors were used in a non-cytotoxic concentration.

### Cell viability and death assay

The cell viability of chemical inhibitors and siRNA were determined with a 3-(4,5-dimethyl-2-thiazolyl)-2,5-diphenyl-2H-tetrazolium bromide (MTT) (Sigma-Aldrich, M2128) assay. In brief, cells were cultured in each well of 96-well plates with varying concentrations of drugs for 24 h, 48 h or 72 h. Following incubation with MTT (0.5 mg/ml) for 4 h, the supernatant of each well was discarded and the insoluble formazan product was dissolved in 100 μl DMSO. The optical density was measured at 570 nm, with a reference wavelength of 630 nm, on a multiscanner autoreader (M450, Bio-rad, USA). The OD570 in control cells was taken as 100% viability. Cell death was assessed by measuring LDH activity released into the culture medium from damaged cells. LDH activity kit (Beyotime, C0016) was used according to the manufacturer's instructions. Unless otherwise specified, the effects of chemical inhibitors or siRNAs on Gyp-L-induced cell death were measured after 24 h treatment.

### Caspase activity measurement

A quantitative enzymatic activity assay was carried out according to the instructions of the manufacturer (Beyotime, China). After treatment with Gyp-L (60 μg/ml) or Z-VAD (50 μM) or ACTD (0.75 μg/ml) for 24 h, ECA-109 cells were washed, collected, lysed, centrifuged, and analyzed for total protein by the Bradford assay. Samples containing 100~300 μg of total protein were incubated with Ac-DEVD-pNA (2 mM), a caspase-3-specific substrate, or with Ac-LEHD-pNA (2 mM), a caspase-9-sepecific substrate, at 37°C for 2 h. Absorbance was measured at 405 nm in a plate reader.

### RNA extraction and quantitative real-time PCR (RT-PCR)

For the mRNA expression level assay, total RNA was extracted with TRIzol reagent (Invitrogen) according to manufacturer's protocol and 1 μg of RNA was then reverse transcribed with a PrimeScript RT reagent kit (TaKaRa). A real-time PCR assay was performed using a Bio-Rad CFX96 real-time PCR system. The results were analyzed with CFX manager software (Bio-Rad). The sequences of primer pairs were shown in supplementary Table S1. Messenger RNA transcription levels were standardized against house-keeping gene *GAPDH*.

### Western blotting

Cells treated with Gyp-L in the presence or absence of inhibitors for indicated times were harvested and lysed in RIPA buffer (Beyotime, P0013B) containing 1 mM PMSF. The lysates were normalized to equal amounts of protein, and were separated by 6–15% gradient SDS-PAGE. After transferring to nitrocellulose membrane, the proteins were probed with the indicated primary antibodies. Detection was conducted by incubation with species-specific HRP-conjugated secondary antibodies. Immunoreactive bands were visualized using ECL blotting detection reagents (Thermo, 34080). GAPDH was used as the loading control.

### Acridine orange and iyso-tracker red staining

AO produces red fluorescence (emission peak at about 650 nm) in lysosomal compartments, and green fluorescence (emission between 530 and 550 nm) in the cytosolic and nuclear compartments. Cell staining with acridine orange (AO, Sigma-Aldrich, A6014) was performed according to published procedures, adding a final concentration of 5 μg/ml for a period of 30 min (37°C, 5% CO_2_). Tumor cells were incubated with indicated compounds for 12 h or starved with HBSS before AO was added. After washing with PBS 3 times, photographs were obtained using a fluorescence microscope (Nikon Ti-u). For LysoTracker Red staining, cells were treated with chemicals (as indicated) for 12 h at 37°C and then incubated with 50 nM LysoTracker Red (Beyotime, C1043) for an additional 1 hour.

### Assays of cell cycle, apoptosis, mitochondrial membrane potential and mitochondrial mass

To determine the effects of Gyp-L on the cell cycle distribution, ECA-109 cells were treated with Gyp-L for 24 h, fixed with 70% (v/v) ethanol, and stained with 50 μg/ml propidium iodide (Sigma, P4170) containing 0.1 mg/ml RNase and 5% Triton X-100 for 30 min at 37°C. The percentage of cells in different phases of the cycle was analyzed using a flow cytometer (BD FACSCalibur, USA) and inbuilt software. For apoptosis analysis, the cells were incubated with various concentrations of Gyp-L for 24 h, harvested, washed, and resuspended in 500 μl of 1× binding buffer containing Annexin V-FITC and PI (BD, 556547) for 10 min at room temperature. Mitochondrial membrane potential and mass were evaluated using the probe JC-1 (Beyotime, C2005), or Mito-Tracker Green (Beyotime, C1048), respectively. Briefly, esophageal cancer cells in each group were harvested, washed twice with cold PBS, centrifuged, and then stained with JC-1 (5 μg/ml) or Mito-Tracker Green (200 nM) for 30 min at 37°C and analyzed using flow cytometry.

### Measurement of intracellular ROS or calcium

Cancer cells treated with indicated compounds for 8 h were stained with 10 μM DCFH-DA (Sigma-Aldrich, D6883) for 30 min. Then ROS generation was determined at 525 nm by flow cytometry. Intracellular calcium concentration was measured by fluorescence microscopy using specific probe Fluo-4/AM (Invitrogen, F-14201). The cells treated with different concentrations of Gyp-L were incubated with 5 μM Fluo-4/AM at 37°C for 60 min and detected by fluorescence microscopy (Nikon Ti-u). Software Image J was used to measure the mean fluorescence intensity.

### RNA interference and transfection

ECA-109 cells were transfected with 2 μg siRNAs against *ATG5*, *ATG7*, *LC3B* (purchased from Genepharma), RAB5 (purchased from Santa Cruz) or control nontargeting siRNA using Lipofectamine 2000 (Invitrogen,11668-019) according to the manufacturer's instructions. Further experiments were performed after transfection 24 h. The sequences of siNRAs were in Supplementary Table S2.

### Autophagosome and iysosome assay

Cultured cells transfected with GFP-LC3 (2 μg) for 24 h, or transfected with Lysosomes-GFP (ThermoFisher, C10596) according to the manufacturer's instructions, were incubated with different chemicals for indicated times. After treatment, the cells were fixed with 4% paraformaldehyde in phosphate-buffered saline (PBS). All the fluorescent images were captured using fluorescence microscope (Nikon Ti-u). At least 50 cells from 5 representative fields were counted in each independent experiment.

For autophagic flux analysis, cells were transfected with mRFP-GFP-LC3 (Invitrogen, 36239) (2 μg) for 24 h before treated with Gyp-L for 12 h. Cells were washed in PBS, fixed in 4% paraformaldehyde-PBS for 15 min. For colocalization analysis, cells were treated with Gyp-L for 24 h and then fixed in 4% paraformaldehyde-PBS for 15 min and permeabilized with 0.1% Triton X-100 for 5 min. The samples were blocked in 5% bovine serum albumin and incubated with anti-LC3B antibody (Cell signaling, 1:1000) and anti-LAMP2 antibody (Abcam, 1:1000). The stained cells were washed and incubated with Alexa Fluor-conjugated secondary antibodies (Cell signaling, 1:1000). Additionally, 1 mg/ml DAPI-PBS was added to label nuclei (15 min). Unless otherwise specified, staining was performed at room temperature for 1 hour. Images were captured with a Zeiss LSM510 Meta confocal system under a 40× oil immersion objective (Carl Zeiss).

### Transmission electronic microscopy assay

ECA-109 cells were treated as indicated and were fixed in 4% glutaraldehyde at 4°C overnight. After dehydration, ultrathin sections were embedded and stained with uranyl acetate/lead citrate. Images were captured under a transmission electron microscope (Tencai G2 20, Netherland).

### Statistical analysis

Data shown in this study are representatives or statistics (mean value ± standard deviation) of the results from at least three independent experiments. Student's two-tailed *t*-test was used for all statistical analysis, with the level of significance set at **p < 0.01; *p < 0.05.

## SUPPLEMENTARY MATERIALS FIGURES AND TABLES



## References

[1] Torre LA, Bray F, Siegel RL, Ferlay J, Lortet-Tieulent J, Jemal A (2015). Global cancer statistics 2012. CA Cancer J Clin.

[2] Shah MA (2015). Update on metastatic gastric and esophageal cancers. J Clin Oncol.

[3] Shahbaz Sarwar CM, Luketich JD, Landreneau RJ, Abbas G (2010). Esophageal cancer: an update. Int J Surg.

[4] Chen N, Karantza V (2011). Autophagy as a therapeutic target in cancer. Cancer Biol Ther.

[5] Karantza-Wadsworth V, Patel S, Kravchuk O, Chen G, Mathew R, Jin S, White E (2007). Autophagy mitigates metabolic stress and genome damage in mammary tumorigenesis. Gene Dev.

[6] Zhang H, Bosch-Marce M, Shimoda LA, Tan YS, Tan YS, Baek JH, Wesley JB, Gonzalez FJ, Semenza GL (2008). Mitochondrial autophagy is an HIF-1-dependent adaptive metabolic response to hypoxia. J Biol Chem.

[7] Degenhardt K, Mathew R, Beaudoin B, Bray K, Anderson D, Chen G, Mukherjee C, Shi Y, Gélinas C, Fan Y, Nelson DA, Jin S, White E (2006). Autophagy promotes tumor cell survival and restricts necrosis inflammation and tumorigenesis. Cancer Cell.

[8] Chen S, Rehman SK, Zhang W, Wen A, Yao L, Zhang J (2010). Autophagy is a therapeutic target in anticancer drug resistance. Biochim Biophys Acta.

[9] Lai K, Killingsworth MC, Lee CS (2014). The significance of autophagy in colorectal cancer pathogenesis and implications for therapy. J Clin Pathol.

[10] Wei MF, Chen MW, Chen KC, Lou PJ, Lin SY, Hung SC, Hsiao M, Yao CJ, Shieh MJ (2014). Autophagy promotes resistance to photodynamic therapy-induced apoptosis selectively in colorectal cancer stem-like cells. Autophagy.

[11] Elgendy M, Sheridan C, Brumatti G, Martin SJ (2011). Oncogenic Ras-induced expression of Noxa and Beclin-1 promotes autophagic cell death and limits clongenic survival. Mol Cell.

[12] Lao Y, Wan G, Liu Z, Wang X, Ruan P, Xu W, Xu D, Xie W, Zhang Y, Xu H, Xu N (2014). The natural compound oblongifolin C inhibits autophagic flux and enhances antitumor efficacy of nutrient deprivation. Autophagy.

[13] Yue W, Hamaï A, Tonelli G, Bauvy C, Nicolas V, Tharinger H, Codogno P, Mehrpour M (2013). Inhibition of the autophagic flux by salinomycin in breast cancer stem-like/progenitor cells interferes with their maintenance. Autophagy.

[14] Livesey KM, Tang D, Zeh HJ, Lotze MT (2009). Autophagy inhibition in combination cancer treatment. Curr Opin Invest Dr.

[15] Mishra BB, Tiwari VK (2011). Natural products: an evolving role in future drug discovery. Eur J Med Chem.

[16] Norberg A, Hoa NK, Liepinsh E, Van Phan D, Thuan ND, Jörnvall H, Sillard R, Ostenson CG (2004). A novel insulin-releasing substance phanoside from the plant Gynostemma pentaphyllum. J Biol Chem.

[17] Zhang GL, Deng JP, Wang BH, Zhao ZW, Li J, Gao L, Liu BL, Xong JR, Guo XD, Yan ZQ, Gao GD (2011). Gypenosides improve cognitive impairment induced by chronic cerebral hypoperfusion in rats by suppressing oxidative stress and astrocytic activation. Behav Pharmacol.

[18] Huyen VT, Phan DV, Thang P, Hoa NK, Ostenson CG (2010). Antidiabetic effect of Gynostemma pentaphyllum tea in randomly assigned type 2 diabetic patients. Horm Metab Res.

[19] Sun H, Zheng Q (2005). Haemolytic activities and adjuvant effect of Gynostemma pentaphyllum saponins on the immune responses to ovalbumin in mice. Phytother Res.

[20] Lin JJ, Hsu HY, Yang JS, Lu KW, Wu RS, Wu KC, Lai TY, Chen PY, Ma CY, Wood WG, Chung JG (2011). Molecular evidence of anti-leukemia activity of gypenosides on human myeloid leukemia HL-60 cells *in vitro* and *in vivo* using a HL-60 cells murine xenograft model. Phytomedicine.

[21] Lu KW, Chen JC, Lai TY, Yang JS, Weng SW, Ma YS, Lin HY, Wu RS, Wu KC, Wood WG, Chung JG (2012). Gypenosides suppress growth of human oral cancer SAS cells *in vitro* and in a murine xenograft model: the role of apoptosis mediated by caspase-dependent and caspase-independent pathways. Integr Cancer Ther.

[22] Hsu HY, Yang JS, Lu KW, Yu CS, Chou ST, Lin JJ, Chen YY, Lin ML, Chueh FS, Chen SS, Chung JG (2011). An experimental study on the antileukemia effects of gypenosides *in vitro* and *in vivo*. Integr Cancer Ther.

[23] Wu YT, Tan HL, Shui G, Bauvy C, Huang Q, Wenk MR, Ong CN, Codogno P, Shen HM (2010). Dual role of 3-methyladenine in modulation of autophagy via different temporal patterns of inhibition on class I and III phosphoinositide 3-kinase. J Biol Chem.

[24] Petiot A, Ogier-Denis E, Blommaart EF, Meijer AJ, Codogno P (2000). Distinct classes of phosphatidylinositol 3′-kinases are involved in signaling pathways that control macroautophagy in HT-29 cells. J Biol Chem.

[25] Sheng Y, Sun B, Guo WT, Zhang YH, Liu X, Xing Y, Dong DL (2013). 3-Methyladenine induces cell death and its interaction with chemotherapeutic drugs is independent of autophagy. Biochem Biophys Res Commun.

[26] Kimura T, Takabatake Y, Takahashi A, Isaka Y (2013). Chloroquine in cancer therapy: a double-edged sword of autophagy. Cancer Res.

[27] Ao X, Zou L, Wu Y (2014). Regulation of autophagy by the Rab GTPase network. Cell Death Differ.

[28] Lee JY, Koga H, Kawaguchi Y, Tang W, Wong E, Gao YS, Pandey UB, Kaushik S, Tresse E, Lu J, Taylor JP, Cuervo AM, Yao TP (2010). HDAC6 controls autophagosome maturation essential for ubiquitin-selective quality-control autophagy. EMBO J.

[29] Lu Y, Dong S, Hao B, Li C, Zhu K, Guo W, Wang Q, Cheung KH, Wong CW, Wu WT, Markus H, Yue J (2014). Vacuolin-1 potently and reversibly inhibits autophagosome-lysosome fusion by activating RAB5A. Autophagy.

[30] Krebs J, Agellon LB, Michalak M (2015). Ca^2+^ homeostasis and endoplasmic reticulum (ER) stress: An integrated view of calcium signaling. Biochem Biophs Res Commun.

[31] Toyoshima C (2009). How Ca^2+^-ATPase pumps ions across the sarcoplasmic reticulum membrane. Biochim Biophys Acta.

[32] Shen M, Wang L, Wang B, Wang T, Yang G, Shen L, Wang T, Guo X, Liu Y, Xia Y, Jia L, Wang X (2014). Activation of volume-sensitive outwardly rectifying chloride channel by ROS contributes to ER stress and cardiac contractile dysfunction: involvement of CHOP through Wnt. Cell Death Dis.

[33] Chen Y, Liu JM, Xiong XX, Qiu XY, Pan F, Liu D, Lan SJ, Jin S, Yu SB, Chen XQ (2015). Piperlongumine selectively kills hepatocellular carcinoma cells and preferentially inhibits their invasion via ROS-ER-MAPKs-CHOP. Oncotarget.

[34] Hasanain M, Bhattacharjee A, Pandey P, Ashraf R, Singh N, Sharma S, Vishwakarma AL, Datta D, Mitra K, Sarkar J (2015). α-Solanine induces ROS-mediated autophagy through activation of endoplasmic reticulum stress and inhibition of Akt/mTOR pathway. Cell Death Dis.

[35] Nguyen PH, Gauhar R, Hwang SL, Dao TT, Park DC, Kim JE, Song H, Huh TL, Oh WK (2011). New dammarane-type glucosides as potential activators of AMP-activated protein kinase (AMPK) from Gynostemma pentaphyllum. Bioorgan Med Chem.

[36] Wu Q, Jang M, Piao XL (2014). Determination by UPLC-MS of four dammarane-type saponins from heat-processed Gynostemma pentaphyllum. Biosci Biotech Bioch.

[37] Piao XL, Wu Q, Yang J, Park SY, Chen DJ, Liu HM (2013). Dammarane-type saponins from heat-processed Gynostemma pentaphyllum show fortified activity against A549 cells. Arch Pharm Res.

[38] Degtyarev M, De Maziere A, Orr C, Lin J, Lee BB, Tien JY, Prior WW, van Dijk S, Wu H, Gray DC, Davis DP, Stern HM, Murray LJ (2008). Akt inhibition promotes autophagy and sensitizes PTEN-null tumors to lysosomotropic agents. J Cell Biol.

[39] Zhao X, Fang Y, Yang Y, Qin Y, Wu P, Wang T, Lai H, Meng L, Wang D, Zheng Z, Lu X, Zhang H, Gao Q (2015). Elaiophylin a novel autophagy inhibitor exerts antitumor activity as a single agent in ovarian cancer cells. Autophagy.

[40] Yoon YH, Cho KS, Hwang JJ, Lee SJ, Choi JA, Koh JY (2010). Induction of lysosomal dilatation arrested autophagy and cell death by chloroquine in cultured ARPE19 cells. Invest Ophthalmol Vis Sci.

[41] Kar R, Singha PK, Venkatachalam MA, Saikumar P (2009). A novel role for MAP1 LC3 in nonautophagic cytoplasmic vacuolation death of cancer cells. Oncogene.

[42] Wasik AM, Almestrand S, Wang X, Hultenby K, Dackland ÅL, Andersson P, Kimby E, Christensson B, Sander B (2011). WIN55 212–2 induces cytoplasmic vacuolation in apoptosis-resistant MCL cells. Cell Death Dis.

[43] Bury M, Girault A, Mégalizzi V, Spiegl-Kreinecker S, Mathieu V, Berger W, Evidente A, Kornienko A, Gailly P, Vandier C, Kiss R (2013). Ophiobolin A induces paraptosis-like cell death in human glioblastoma cells by decreasing BKCa channel activity. Cell Death Dis.

[44] Sperandio S, Poksay K, de Belle I, Lafuente MJ, Liu B, Nasir J, Bredesen DE (2004). Paraptosis: mediation by MAP kinases and inhibition by AIP-1/Alix. Cell Death Differ.

[45] Sperandio S, de Belle I, Bredesen DE (2000). An alternative nonapoptotic form of programmed cell death. Proc Natl Acad Sci U S A.

[46] Lee WJ, Chien MH, Chow JM, Chang JL, Wen YC, Lin YW, Cheng CW, Lai GM, Hsiao M, Lee LM (2015). Nonautophagic cytoplasmic vacuolation death induction in human PC-3M prostate cancer by curcumin through reactive oxygen species-mediated endoplasmic reticulum stress. Sci Rep.

[47] Yoon MJ, Lee AR, Jeong SA, Kim YS, Kim JY, Kwon YJ, Choi KS (2014). Release of Ca^2+^ from the endoplasmic reticulum and its subsequent influx into mitochondria trigger celastrol-induced paraptosis in cancer cells. Oncotarget.

[48] Williams JA, Hou Y, Ni HM, Ding WX (2013). Role of intracellular calcium in proteasome inhibitor-induced endoplasmic reticulum stress autophagy and cell death. Pharm Res.

[49] Williams A, Sarkar S, Cuddon P, Ttofi EK, Saiki S, Siddiqi FH, Jahreiss L, Fleming A, Pask D, Goldsmith P, O'Kane CJ, Floto RA, Rubinsztein DC (2008). Novel targets for Huntington's disease in an mTOR-independent autophagy pathway. Nat Chem Biol.

[50] Ganley IG, Wong PM, Gammoh N, Jiang X (2011). Distinct autophagosome-lysosomal fusion mechanism revealed by thapsigargin-induced autophagy arrest. Mol Cell.

[51] Engedal N, Torgersen ML, Guldvik IJ, Barfeld SJ, Bakula D, Sætre F, Hagen LK, Patterson JB, Proikas-Cezanne T, Seglen PO, Simonsen A, Mills IG (2013). Modulation of intracellular calcium homeostasis blocks autophagosome formation. Autophagy.

