# Supplementary material for: Gypenoside L inhibits autophagic flux and induces cell death in human esophageal cancer cells through endoplasm reticulum stress-mediated Ca2+ release
Source: Oncotarget. 2016 Jun 18;7(30):47387–402. doi: 10.18632/oncotarget.10159 (PMC5216949; doi:10.18632/oncotarget.10159)
Supplement: Supplementary file 1 [file oncotarget-07-47387-s001.pdf]

Gypenoside L inhibits autophagic flux and induces cell death in human esophageal cancer cells through endoplasm reticulum stress-mediated Ca^{2+} release

Supplementary Materials

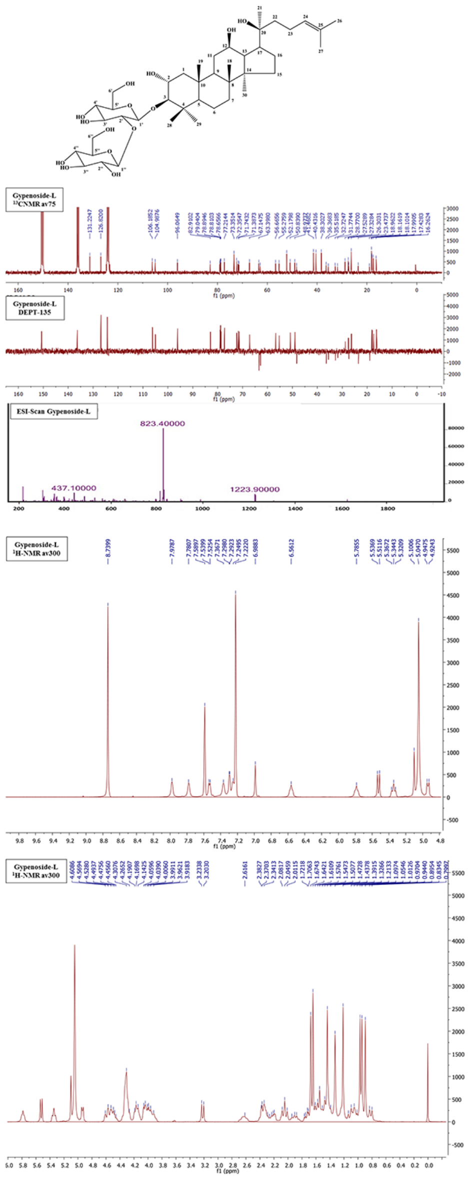

Supplementary Figure S1: Isolation and characterization of Gyp-L. The total saponins of *G. pentaphyllum* were kindly provided by William chi-shing Tai (Hong Kong Baptist University). A portion of total saponins (350 g) was firstly subjected to column chromatography over silica gel (6000 g, 300−400 mesh) and eluted with the isocratic gradient solvent system of $CHCl_3$, methanol and water (26:8:1) to yield twenty-four major fractions (Fraction 1-24). Fraction 14 (4.7g) was then subjected to column chromatography over ODS (239.48g,100-200 mesh) and eluted with the isocratic gradient solvent system of 40% CH3CN in water to yield four major components (**A**–**D**). (B) (265 mg) was identified as gypenoside L by ^1H, ^{13}C NMR and LC-MS. ^{13}C NMR (75 MHz, C_5D_5N): δ 48.24 (C1), 67.15 (C2), 96.06 (C3), 41.47 (C4), 56.67 (C5), 18.96 (C6), 35.52 (C7), 40.43 (C8), 50.84 (C9), 38.30 (C10), 32.72 (C11), 71.74 (C12), 48.97 (C13), 52.18 (C14), 31.77 (C15), 27.52 (C16), 55.28 (C17), 16.26 (C18), 18.16 (C19), 73.35 (C20), 7.32 (C21), 36.37 (C22), 23.47 (C23), 126.82 (C24), 131.22 (C25), 26.30 (C26), 18.10 (C27), 28.77 (C28), 17.99 (C29), 17.43 (C30), 106.19 (C1′), 82.91 (C2′), 78.89 (C3′), 72.35 (C4′), 78.66 (C5′), 63.40 (C6′), 104.99 (C1″), 77.21 (C2″), 79.04 (C3″), 71.39 (C4″), 78.81 (C5″), 62.82 (C6″). ESIMS: *m/z* 823.40 $[M+Na]^+$ (caclcd for $C_{42}H_{72}O_{14}$,800.49).

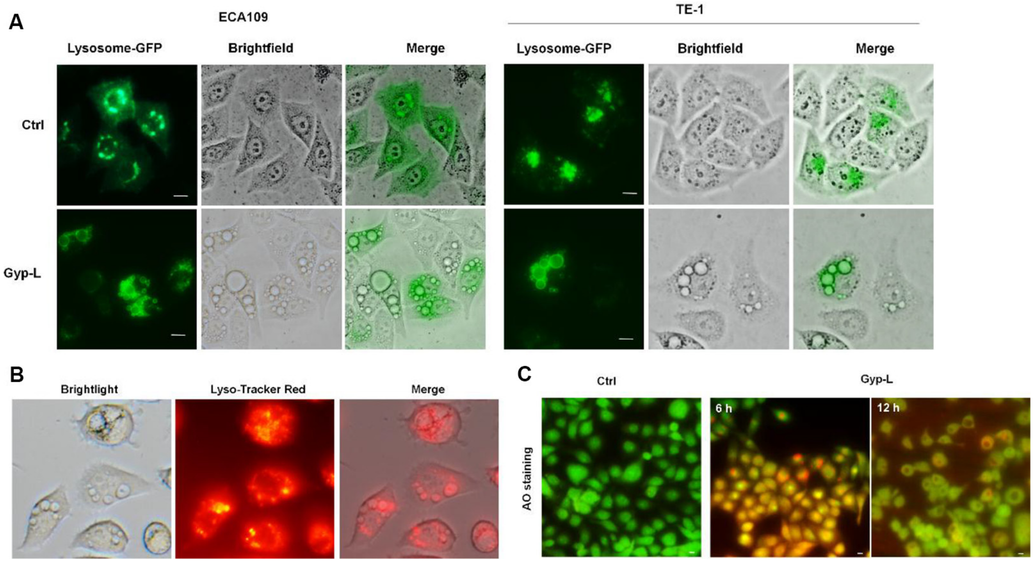

Supplementary Figure S2: Gyp-L induces lysosomal swelling and dysfunction. (**A**) ECA-109 or TE-1 cells were transfected with lysosome-GFP (LAMP1-GFP) for 24 h before treated with Gyp-L for 24 h. Images were captured using fluorescence microscope. Scale bar: 20 µm. (**B**) Lyso-Tracker Red staining. Cells treated with Gyp-L for 24 h were stained with 50 nM Lyso-Tracker Red. (**C**) AO staining assay. ECA-109 cells were treated with Gyp-L (60 µg/ml) for 6 h or 12 h, and cells were washed, stained with AO (5 µg/ml) for 30 min. Fluorescence images were captured by fluorescence microscopy and representative photomicrographs were shown. Scale bar: 20 µm.

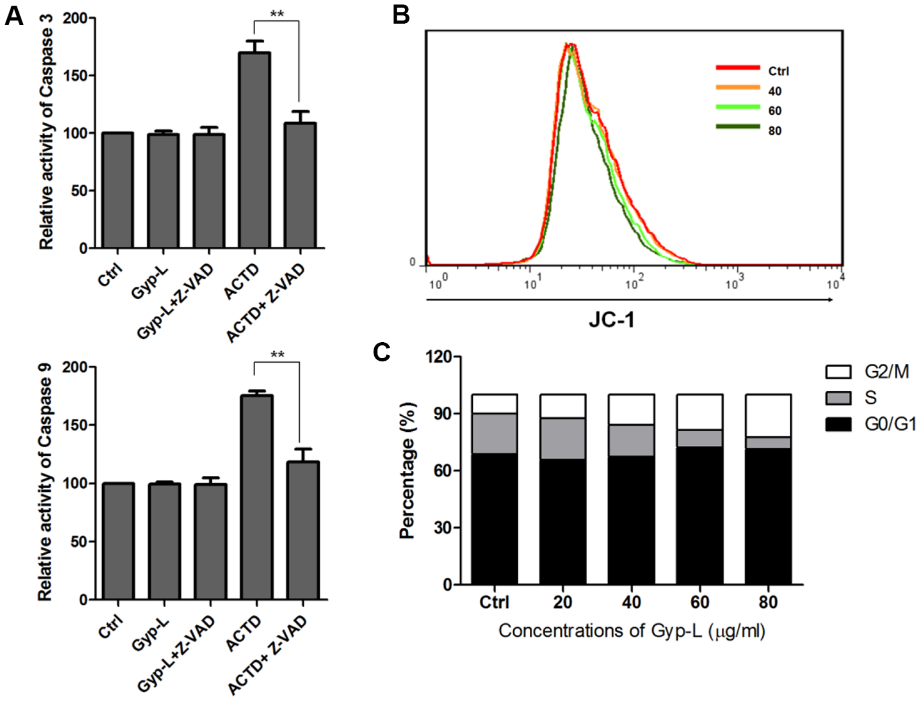

Supplementary Figure S3: Gyp-L induces nonapoptotic cell death. (**A**) After ECA-109 cells treated with Gyp-L (80 µg/ml) in the presence or absence of Z-VAD for 24 h, total cell lysates were extracted to measure the activity of caspase 3 or caspase 9. Dactinmycin (ACTD) (0.75 µg/ml), a mRNA synthesis inhibitor, was used as a positive control. (**B**) ECA-109 cells were treated with Gyp-L for 12 h and mitochondrial membrane potential was measured by Flow cytometry using JC-1 staining. (**C**) Effects of Gyp-L on cell cycle distribution. ECA-109 Cells were treated with 0, 20, 40, 60 and 80 µg/ml Gyp-L for 24 h, fixed in 70% ethanol, stained with PI, and cell cycle distribution was assessed by flow cytometry. The percentage of cells in each cell cycle phase is indicated as the mean ± SD of three independent experiments.

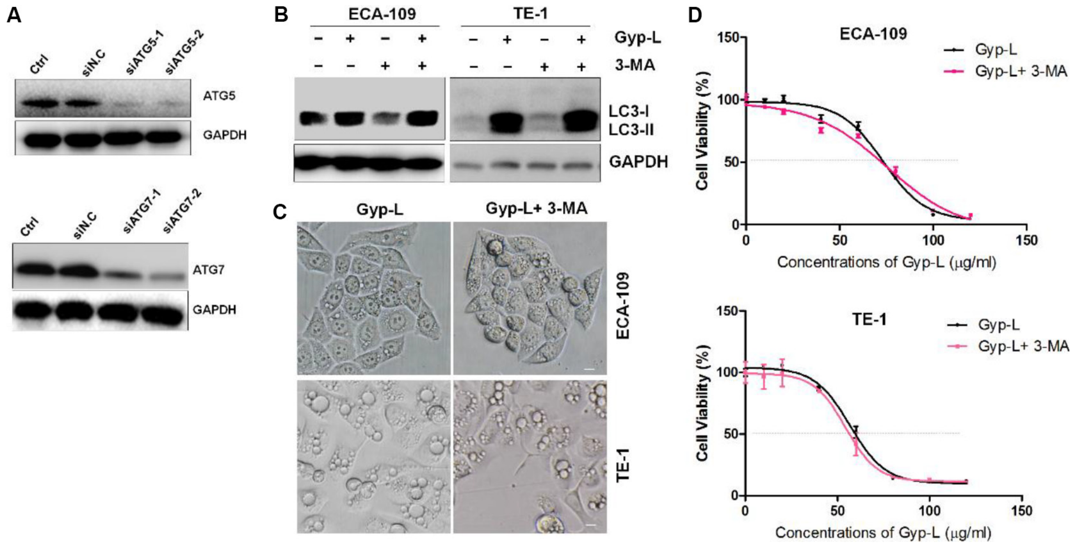

Supplementary Figure S4: Effect of 3-MA on Gyp-L-induced cell death. (**A**) Efficiency of knockdown of ATG5 and ATG7. (**B**) ECA-109 and TE-1 cells were treated with Gyp-L in the presence or absence of 3-MA (10 mM) for 12 h, and cell lysates were subjected to western blot for LC3-II and p62. (**C–D**) 3-MA dose not inhibit Gyp-L-induced cytoplasmic vacuoles and cell death. ECA-109 or TE-1 cells were treated with Gyp-L and 3-MA for 24 h and cell viability was measured with the MTT assay.

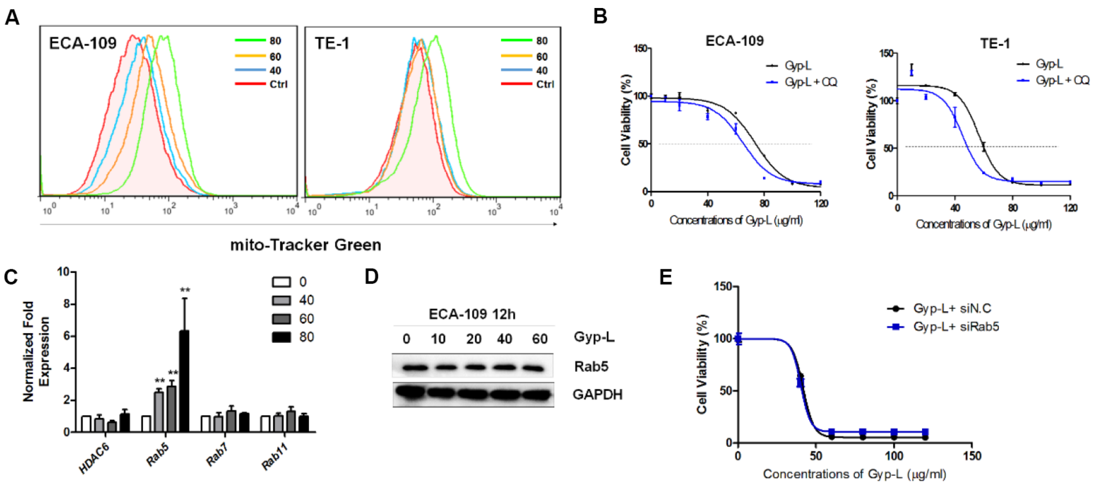

Supplementary Figure S5: Gyp-L inhibits autophagic flux. (**A**) Effect of Gyp-L on mitochondrial mass. (**B**) CQ rendered cancer cells more sensitive to the cytotoxicity of Gyp-L. ECA-109 or TE-1 cells were treated with Gyp-L in the presence or absence of CQ for 24 h. (**C**) Expression of several lysosomal positioning genes. ECA-109 cells were treated with Gyp-L (60 μg/ml) for 12 h and the total RNA was extracted. A quantitative real-time PCR (RT-PCR) was performed to determine the mRNA levels of *RAB5*, *RAB7*, *RAB11* and *HDAC6*. Data presented are representative of three independent experiments. (**D**) ECA-109 cells were treated with Gyp-L for 12 h and cell lysates were subject to western blot for RAB5 and GAPDH. (**E**) ECA-109 cells were transfected with siRNA targeting RAB5 for 24 before treated with Gyp-L for another 24 h. Cell viability was measured with the MTT assay.

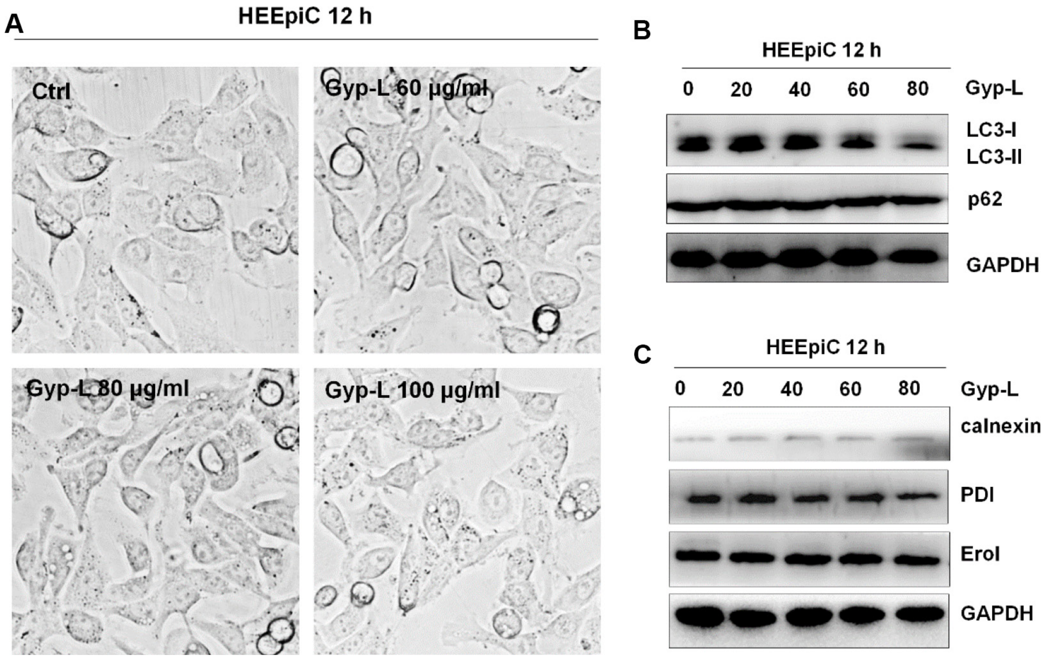

Supplementary Figure S6: Normal HEEpiC cells are protected from Gyp-L-induced cytotoxic effects. (**A**) HEEC cells were treated with Gyp-L for 12 h. (**B**) Western blots of total cell lysates on the expression of LC3-II and p62. (**C**) Western blots of total cell lysates on the expression of ER stress-related proteins.

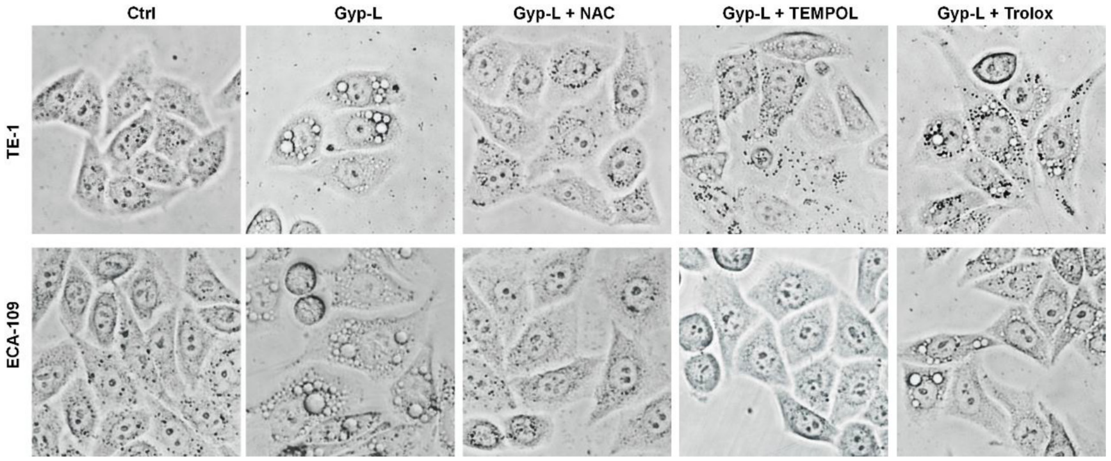

Supplementary Figure S7: Effect of ROS scavenger on Gyp-L-induced cytoplasmic vacuolation. ECA-109 or TE-1 cells were treated with Gyp-L in the presence or absence of NAC (5 mM), TEMPOL (2 mM), Trolox (1 mM) for 12 h.

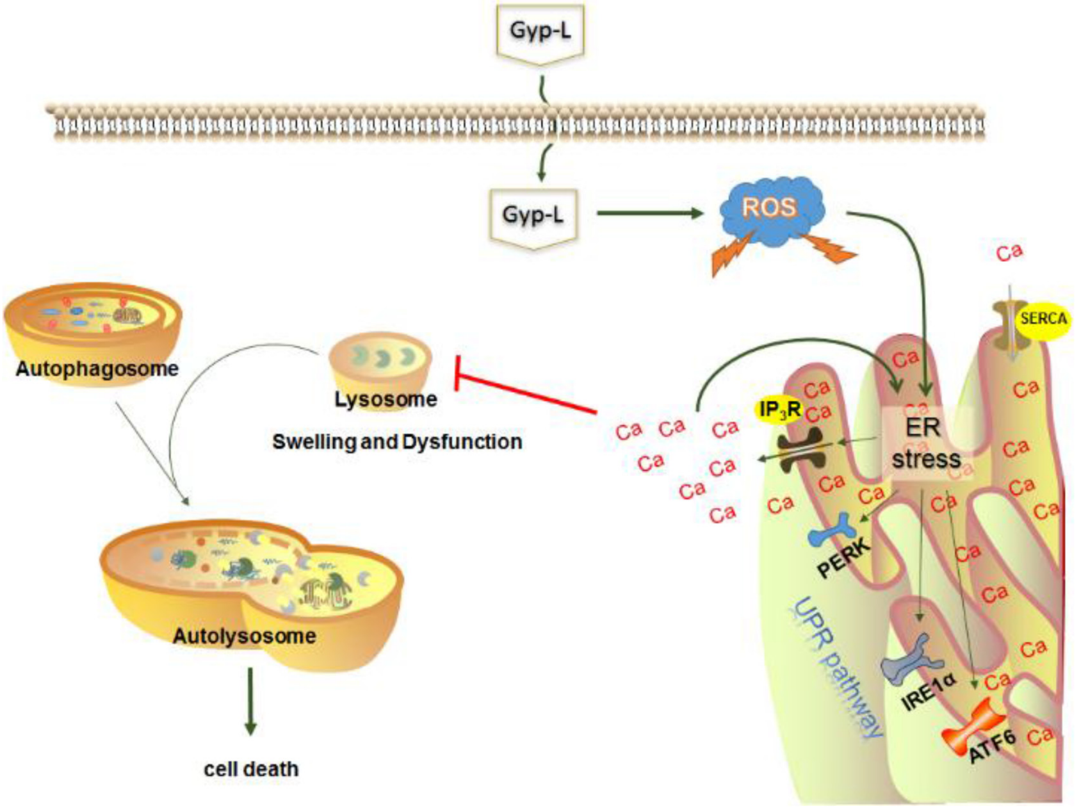

Supplementary Figure S8: Proposed mechanism by which Gyp-L inhibits autophagic flux and induces cell death in esophageal cancer cells. Gyp-L initially increases the intracellular ROS production, and subsequently causes the ER stress, UPR pathway activation, and Ca^{2+} release from ER. Then the increased cytoplasm Ca^{2+} induces the swelling and dysfunction of lysosomes, leading to the inhibition of autophagic flux and finally lysosome-associated cell death.

Supplementary Table S1: primers of RT-PCR

Genes	Sequences
HDAC6	Forward: CCACAACCAGGCAGGCAGCGAAGAAG
	Reverse: ATCCATCCCTTGCAGTCCCACG
SERCA	Forward: TCAAGGGAGTTCAATGTGCCCTCT
	Reverse: AAGGTGGCATGACCAGCATAGACT
RAB5	Forward: ACCACCGCCATAGATACACTCTCA
	Reverse: ACTAGGCTTGATTTGCCAACAGCG
RAB7	Forward: AGTTCCCTGGAACCAGAACTTGGA
	Reverse: TGTGACTAGCCTGTCATCCACCAT
RAB11	Forward: GGAAAGCAAGAGCACCATTGGAGT
	Reverse: TTTGTAGAGTCTAGGGCCGAAGTTTC
IP3R1	Forward: TGACGAGAACCTGCCCTAT
	Reverse: TCCTTTCGCCATCTTGCT
GAPDH	Forward: AGCCTCAAGATCATGAGCAATG
	Reverse: TCACGATACCAAAGTTGTCATGGA

Supplementary Table S2: siRNA sequences

Gene	Sense (5′–3′)	Antisense (5′–3′)
LC3-1	AGCUGUGGAUGAUCCACGU	ACGUGGAUCAUCCACAGCU
ATG5-1	GGAUGCAAUUGAAGCUCAU	AUGAGCUUCAAUUGCAUCC
ATG5-2	GUUGGUCAAAGACCAGAUA	UAUCUGGUCUUUGACCAAC
ATG7-1	CCCUGUACUCCUCAACAAG	CUUGUUGAGGAGUACAGGG
ATG7-2	GUACCACUUCUACUAUUGG	CCAAUAGUAGAAGUGGUAC
